# Detection of anabolic steroids *via* cyclodextrin-promoted fluorescence modulation†

**DOI:** 10.1039/d0ra03485a

**Published:** 2020-07-01

**Authors:** Anna Z. Haynes, Mindy Levine

**Affiliations:** Ariel University, Department of Chemical Sciences 65 Ramat HaGolan Street Ariel Israel mindy.levine@gmail.com mindyl@ariel.ac.il; University of Rhode Island, Department of Chemistry 140 Flagg Road Kingston RI 02881 USA

## Abstract

Reported herein is the detection of anabolic steroids through the use of cyclodextrin-promoted interactions between the analyte of interest and a high quantum yield fluorophore, which lead to measurable, analyte-specific changes in the fluorophore emission signal. By using a variety of β-cyclodextrin derivatives (unmodified β-cyclodextrin, methyl-β-cyclodextrin, and 2-hydroxypropyl-β-cyclodextrin) in combination with high quantum yield fluorophore rhodamine 6G, we detected five anabolic steroid analytes with 100% differentiation between structurally similar analytes and micromolar level limits of detection. Overall, these results show significant potential in the development of practical, fluorescence-based steroid detection devices.

## Abstract 2

Reported herein is the detection of anabolic steroids through the use of cyclodextrin-promoted interactions between the analyte and a high quantum yield fluorophore, which leads to analyte-specific changes in the fluorophore emission signal.

## Graphical abstract

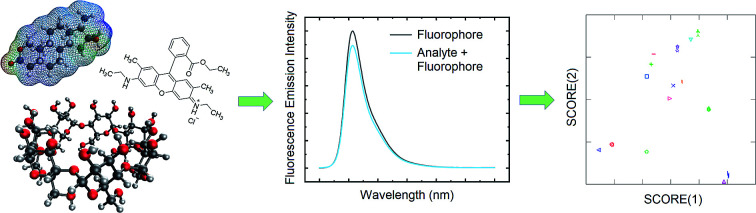

## Introduction

The detection of steroids using sensitive, selective, and portable methods is of significant interest in a variety of contexts, particularly in sporting scenarios in which illegal anabolic steroid use has been reported.^1^ Such illegal use has been increasing in recent years at all competition levels, including in elementary school athletic programs, and the lack of effective methods for steroid detection means that the usage is likely to continue to increase unless a rapid, sensitive, selective, and easy-to-use method is developed.^2^ Detection methods that currently exist suffer from a variety of drawbacks,^3^ including the need for expensive laboratory instrumentation and significant sample preparation prior to analysis, that make them impractical for on-site usage at sporting events.^4^ Although fluorescence-based detection of steroids has been reported, such detection generally requires chromatography,^5^ additional additives,^6^ or expensive instrumentation,^7^ which adds significant time and cost to the analysis. As such, a new method is needed.

New approaches to chemical detection have been developed in recent years, and include the use of array-based detection methods as artificial “noses” and “tongues” for pattern-based chemical detection. Such approaches have been pioneered by the research groups of Eric Anslyn,^8^ Kenneth Suslick,^9^ Vincent Rotello,^10^ and Uwe Bunz,^11^ among others.^12^ Array-based analysis has led to remarkably high levels of chemical selectivity and accuracy in identification of unknown compounds, by using principles that are similar to those used by the human tongue^13^ and nose.^14^ Such approaches are inherently supramolecular in nature, as the binding of the target analyte with the array of receptors relies on a variety of non-covalent interactions to achieve effective and selective complexation.^15^ Moreover, steroid sensing has also been accomplished *via* supramolecular complexation, using cucurbiturils to achieve effective steroid-induced changes in the sensor signal response.^16^

One related approach to a new detection method has been developed by the Levine group in recent years, and relies on the use of cyclodextrin as a supramolecular scaffold to promote proximity-induced interactions between the analyte of interest and a high quantum yield fluorophore that leads to effective fluorescence detection.^17^ Such detection has been demonstrated for a variety of analytes, including polycyclic aromatic hydrocarbons,^18^ bisphenols,^19^ pesticides,^20^ and alcohols,^21^ and in a variety of contexts, including in the complex matrices of human breast milk,^22^ urine,^10^ and saliva.^23^ High detection sensitivity and selectivity has also been demonstrated, and is maintained even in the aforementioned complex biological matrices. The fact that the read-out signal of such sensors is a rapid and measurable change in the fluorescence emission signal means that the system is easily translatable for on-site measurements. The use of such a fluorescence-based detection system for steroid detection has not been reported to date, despite the advantages of the proposed system. Moreover, significant literature precedent indicates that steroids form strong supramolecular complexes with cyclodextrin,^24^ some of which have been used for electrochemically-driven steroid detection,^25^ which increases the likelihood of an effective cyclodextrin-mediated steroid detection strategy.

**Fig. 1:**
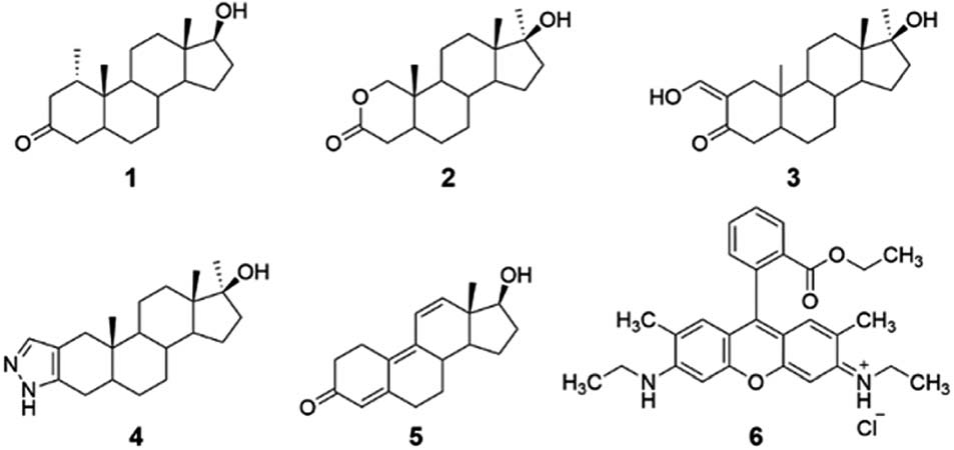
Structure of anabolic analytes investigated herein: [mesterolone (compound 1); oxandrolone (compound 2); oxymetholone (compound 3); stanozolol (compound 4); and trenbolone (compound 5)]; and Rhodamine 6G as the high-quantum yield fluorophore (compound 6).

Reported herein is the cyclodextrin-promoted fluorescence detection of five anabolic steroids: mesterolone, oxandrolone, oxymetholone, trenbolone, and stanozolol (compounds 1–5, Fig. 1). When these analytes are combined with cyclodextrin hosts and a high quantum-yield fluorophore rhodamine 6G (compound 6), highly sensitive, analyte-specific changes in the fluorescence emission of the fluorophore results. Particularly promising results were seen using β-cyclodextrin (β-CD) and its derivatives, methyl-β-cyclodextrin (Me-β-CD) and 2-hydroxypropyl-β-cyclodextrin (2-HP-β-CD), as the supramolecular hosts, with limits of detection as low as 0.05 μM obtained and 100% success in separating the signals obtained from the five steroid analytes using linear discriminant analysis. Overall, these results provide a promising proof-of-concept for successful steroid detection using cyclodextrin-promoted fluorescence changes. Although these results do not include testing in biological matrices, they provide important proof-of-concept data that can eventually be used for on-site detection of illegal doping in competitive sporting scenarios.

## Experimental

### Materials and methods

The anabolic steroid analytes, buffer chemicals, Rhodamine 6G, tetrahydrofuran (THF), and dimethyl sulfoxide (DMSO)-*d*_6_ were obtained from Sigma-Aldrich Chemical Company and the cyclodextrins were obtained from Tokyo Chemical Industry. All chemicals were used as received. All fluorescence measurements were performed using a Shimadzu RF 6000 spectrophotometer. The excitation and emission slit widths were set to 3.0 nm. All fluorescence spectra were integrated *vs.* wavenumber on the *X*-axis using OriginPro 2019 Version 9.60. All arrays were generated using SYSTAT Version 13.1. NMR studies were conducted using a Bruker 400 MHz NMR spectrometer and spectra were analyzed using MestReNova 14.1 software. Electrostatic potential map models were generated using Spartan' 18 software.

### Fluorescence modulation experiments

In six 15 mL glass vials, 100.0 μL of a fluorophore 6 solution (0.1 mg mL^−1^) in THF, 2.00 mL of a 10 mM cyclodextrin solution in citrate buffer (measured at a pH of 6.1 and remaining consistent throughout the experiment) and 0.400 mL of a 0.1 M citrate buffer were combined. These solutions were left to stabilize for 48 hours in a dark drawer and then transferred into a quartz cuvette. To the first cuvette, 5.00 μL of analyte 1 was added, in the second cuvette, 5.00 μL of analyte 2 was added, and so on until analyte 5 was added to a cuvette. Of note, additions of 5 μL analyte correspond to a 2.0 ppm final concentration, addition of 10 μL of analyte corresponds to a 3.98 ppm final concentration, and addition of 20 μL of analyte corresponds to a final concentration of 7.94 ppm. 5.00 μL of THF was added to the last cuvette as a control. The solutions were excited at 490 nm and the fluorescence spectra were recorded from 500–800 nm. This was repeated for each cyclodextrin solution as well as in a cyclodextrin-free control, where citrate buffer (pH 6.1) was used instead of a cyclodextrin solution. The procedure was then repeated using 10.00 μL and 20.00 μL additions of analyte instead of 5.00 μL additions. All measured fluorescence spectra were integrated *vs.* wavenumber on the *X*-axis. The fluorescence modulation of each analyte was determined using eqn (1), below:

1 Fluorophore ratio = Fl_analyte_/Fl_blank_

where Fl_analyte_ represents the integrated fluorescence emission of the fluorophore in the presence of the analyte, and Fl_blank_ represents the integrated fluorescence emission of the fluorophore in the absence of analyte. All trials were repeated four times and the reported modulation values represent the average of those repeated trials with the standard deviation values from those trials included as well.

### Limit of detection experiments

The limit of detection (LOD), defined as the lowest concentration of the analyte that can be detected, and the limit of quantification (LOQ), defined as the lowest concentration of analyte that can be reliably quantified, were calculated. The limit of detection and quantification experiments were obtained following literature-reported procedures.^26^ These experiments were done with sequential 5 μL additions of analyte, to the same initial solution matrix described in the fluorescence modulation experiments (see ESI† for more details).

### Array generation experimental details

Arrays were generated using SYSTAT 13 statistical computing software with the following software settings: (a) linear discriminant analysis, (b) grouping variable: analytes, (c) predictors: β-cyclodextrin (β-CD), methyl-β-cyclodextrin (Me-β-CD), and 2-hydroxypropyl-β-cyclodextrin (2-HP-β-CD), and (d) long-range statistics: Mahal. These experiments were then repeated using only two predictors instead of all three, and the results of array-based analysis for each pair of predictors are also reported herein.

### Computational experiments

Spartan' 18 was used to calculate the equilibrium values of the analytes in their ground-state electric potential surfaces using a semi-empirical PM3 model for each analyte.

## Results and discussion

### System design

The selected anabolic steroids were chosen because they are included in the lists of banned and misused substances in athletic competitions.^27^ Fluorophore 6 was chosen due to its efficiency in fluorescence based detection systems, and in particular due to its documented ability to associate with β-cyclodextrin^28^ and its derivatives,^29^ which has been reported both by this group^30^ and others.^31^ Cyclodextrins were chosen as the supramolecular hosts in this study because of their known ability to bind a variety of guest molecules, including steroids,^32^ due to their ability to engage in favorable non-covalent intermolecular interactions including hydrogen bonding, hydrophobic interactions in the interior cavity, and van der Waals interactions.^33^ β-cyclodextrin in particular has a well-documented ability to bind a variety of hydrophobic analytes, including steroids with significant structural similarity to analytes 1–5.^34^ α-Cyclodextrin and γ-cyclodextrin, by contrast, with average cavity diameters of 5.2 Å and 8.4 Å, respectively, were determined to have non-ideal size matches with analytes of average diameters of 5.6 Å.^35^ The two derivatives of β-cyclodextrin selected, methyl-β-cyclodextrin and 2-hydroxypropyl-β-cyclodextrin, have some similarities to the unmodified analogue, but their notable differences in hydrophobicity, solubility, and steric accessibility means that the binding of analytes 1–5 in these derivatives is expected to differ from binding in the unmodified β-cyclodextrin host.^36^ As a result, the use of the three supramolecular hosts was expected to lead to selectivity in array-based linear analysis, an expectation that was successfully borne out in experiments (*vide infra*).^37^

### Fluorescence modulation experiments

**Fig. 2:**
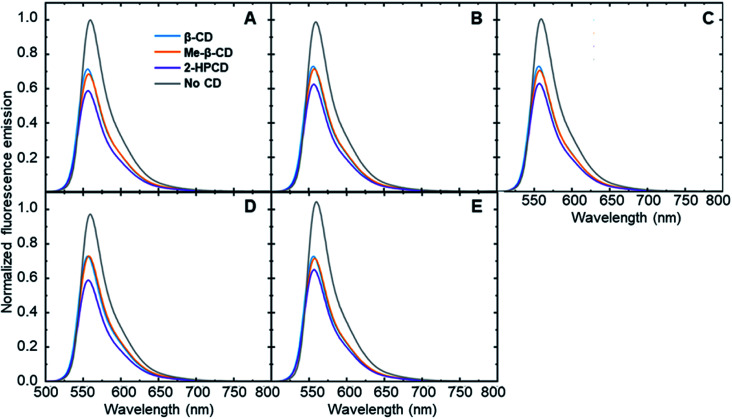
Fluorescence modulation of fluorophore 6 in the presence of all hosts induced by the addition of (A) analyte 1 (B) analyte 2 (C) analyte 3 (D) analyte 4 and (E) analyte 5 (final concentration of each analyte: 8 ppm). Curves were normalized so that the highest fluorescence intensity for each panel was set to 1.0.

In order to show that each steroid is capable of inducing an analyte-specific change in fluorescence emission, fluorescence modulation experiments were performed with each analyte–cyclodextrin combination, together with control trials run in the absence of a cyclodextrin host. Small amounts of the steroids in tetrahydrofuran were added to a solution of cyclodextrin and fluorophore 6 that had been left to stabilize. The fluorescence emission of the fluorophore was measured at an excitation wavelength of 490 nm and compared to the fluorescence emission spectra of the fluorophore after the addition of 5 μL, 10 μL, and 20 μL of the analytes. The degree of fluorescence modulation of the curves was reported as the ratio of the integrated emission of the fluorophore in the absence of analyte to the integrated emission of the fluorophore in the presence of analyte, calculated according to eqn (1). The results of the analyte-induced fluorescence modulation obtained after adding 20 μL of steroid solution are summarized in Table 1 and Fig. 2.

**Table 1:** Fluorescence ratios obtained for the addition of analytes 1–5 with various cyclodextrins in the presence of fluorophore 6^a^

Analyte	β-CD	Me-β-CD	2-HP-β-CD	No CD
1	0.948	0.932	0.892	0.994
2	0.974	0.966	0.947	0.987
3	0.978	0.954	0.955	1.004
4	0.975	0.986	0.952	0.972
5	0.975	0.969	0.988	1.036

a All values were obtained after the addition of 20 μL of steroid solution. The results were calculated using eqn (1) and represent an average of at least four trials.

These results show that adding steroid analytes to a cyclodextrin–fluorophore complex in primarily aqueous solutions leads to measurable, analyte-specific fluorescence changes. In the trials where cyclodextrin was present, each analyte induced a unique response, showing a key role for the cyclodextrin in enabling such specificity to occur. In contrast, the cyclodextrin-free controls showed minimal fluorescence modulation, with less differences between the analytes. Moreover, in the vast majority of cases, the addition of steroid analytes caused a decrease in the observed fluorophore emission, represented by a fluorescence modulation value less than 1. This decrease is likely due to the fact that the steroids cause a displacement of the fluorophore from the cyclodextrin cavity, which increased the availability of non-radiative decay pathways and in turn led to a decrease in the observed fluorescence emission. Analyte 5 in the absence of cyclodextrin represents a notable exception to this trend, and may be a result of the reported fluorescence activity of analyte 5,^38^ which can interfere with the fluorophore emission signal.

**Fig. 3:**
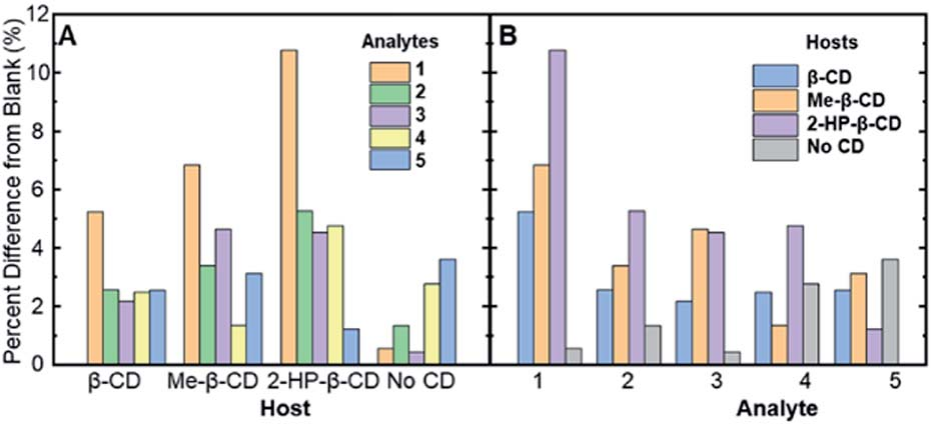
The absolute values of the percent difference values from Table 2, grouped by (A) the various cyclodextrin hosts and (B) the analytes (compounds 1–5) in solution.

Another way of viewing these analyte-specific fluorescence emission changes is through calculating the percent differences induced by each analyte when compared to the blank (*i.e.* analyte-free) sample in the same host solution. These values were calculated according to eqn (2), below:

2 Percent (%) difference = (1 − Fl_ratio_) × 100%

where Fl_ratio_ is the value found in Table 1. The percent differences obtained for each analyte in each host-fluorophore solution were calculated, and the results are summarized in Table 2 and Fig. 3, below.

**Table 2:** Percent (%) difference between the fluorescence emission without analyte and the fluorescence emission after the addition of analyte^a^

Analyte	β-CD	Me-β-CD	2-HP-β-CD	No CD
1	5.24	6.83	10.8	0.566
2	2.56	3.39	5.27	1.35
3	2.16	4.64	4.53	−0.44^b^
4	2.48	1.36	4.75	2.76
5	2.54	3.11	1.23	−3.61^b^

a Percent difference determined after 8 ppm of each analyte was added, using the fluorescence modulation ratios determined in Table 1 and entered into eqn (2).

b Negative values represent a situation where the fluorescence signal of the fluorophore in the presence of analyte was greater than the fluorescence signal of the fluorophore in the absence of analyte.

Fig. 3A represents the percent difference values organized by the different supramolecular hosts, allowing for visualization of which host facilitates the greatest degree of modulation. Among the various hosts that were investigated, 2-HP-β-CD showed the greatest analyte-induced fluorescence modulation. This result is likely due to the fact that unmodified β-cyclodextrin has a highly rigid structure that facilitates the formation of self-assembled architectures in aqueous solution.^39^ Such architectures, in turn, can hinder the effective formation of association complexes, such as those required for steroid-induced fluorescence modulation.^40^ Modified β-cyclodextrin derivatives, including 2-HP-β-CD and Me-β-CD, have markedly more flexible structures, which means less self-assembled architectures and greater accessibility of the cyclodextrins for the target association complexes and steroid-induced fluorescence modulation.^41^ Variability between analyte-induced responses was also seen using Me-β-CD, which is likely due to the hydrophobicity of the cyclodextrin host that facilitates increased analyte–cyclodextrin interactions and greater variations in the observed modulation values. In contrast to the two substituted β-cyclodextrin hosts, unmodified β-cyclodextrin showed the least analyte-induced fluorescence modulation, which is likely due to the factors mentioned above, which limit the intermolecular interactions between the cyclodextrin and the analyte and the resulting analyte-induced fluorescence modulation. Of note, control trials without a cyclodextrin host displayed the lowest average fluorescence modulation, highlighting the critical role of cyclodextrin in acting as an effective supramolecular scaffold to promote analyte-specific interactions with the high quantum yield fluorophore.

**Fig. 4:**
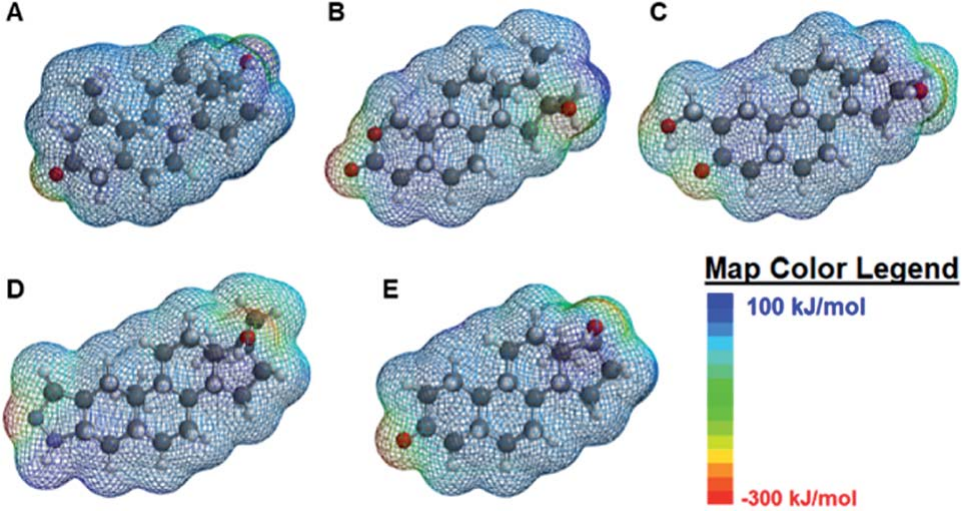
Spartan-calculated electrostatic potential maps of (A) analyte 1 (B) analyte 2 (C) analyte 3 (D) analyte 4 and (E) analyte 5. The areas in the dark blue shade corresponds to electron-deficient, non-polar regions of the model and scales to the red regions, corresponding to the electron-rich, polar regions of the molecule. Color code for the molecular models: dark grey: carbon (C), light grey: hydrogen (H), red: oxygen (O), and light purple: nitrogen (N).

When the degree of fluorescence modulation was measured by analyte (Fig. 3B), analyte 1 was able to induce the highest degree of fluorescence modulation in the cyclodextrin hosts, whereas analyte 5 induced the lowest degree of modulation. Moreover, analytes 2–4 have a similar, intermediate level of analyte-induced modulation. Differences in analyte-induced responses can be explained with the aid of computed electrostatic potential maps, which show the molecular charge distributions (Fig. 4), and the quantitative electrostatic surface values (Table 3), which show minimum and maximum electrostatic potentials, dipole moments and polarized surface areas.

**Table 3:** Quantitative values calculated from the electrostatic potential maps of the steroids in Spartan 18'

Analyte	Minimum electrostatic potential^a^ (kJ mol^−1^)	Maximum electrostatic potential^b^ (kJ mol^−1^)	Dipole moment^c^ (D)	Polar surface area^d^ (Å^2^)
1	−262.3	114.2	2.11	34.117
2	−301.9	114.4	5.14	41.129
3	−262.4	114.1	2.69	48.988
4	−348.4	123.7	3.69	43.486
5	−257.9	100.7	2.23	34.214

a Corresponds to the red, electron-rich regions of the electrostatic potential maps.

b Corresponds to the dark blue, electron-poor regions of the electrostatic potential maps.

c D: abbreviation of the unit debye, dipole moment resulting from two charges of opposite sign but an equal magnitude of 10^−10^ statcoulomb.

d Polar areas that occur due to electronegative elements and hydrogen atoms attached to them in a molecule.

Overall, the main differences in the analyte structures occurs in the polar, electron-rich region on the left side of the molecule, the region where the minimum electrostatic potential fluctuates. This potential is used to gauge how reactive different regions of the molecule are, as well as to provide insight into non-covalent intermolecular interactions.^42^ For example, Fig. 4A shows the structure of mesterolone (analyte 1) which has one hydroxyl group extending from the carbon ring on the left side of the molecule, while Fig. 4B illustrates oxandrolone (analyte 2), which has an ester in the same region. The minimum electrostatic potential energy increases in magnitude from analyte 1 to analyte 2, suggesting that analyte 2 has greater reactivity towards other species. In analyte 2, this change in structure also results in an increased polar surface area, defined as the area on the surface of a molecule that is affected by the charge of electronegative atoms and elements such as nitrogen and oxygen atoms as well as any hydrogens that are bonded to these elements.^43^ There is also a much higher dipole moment, defined as the magnitude of the sum of net charge in a molecule based on the combination of nuclear and electron charges, which provides additional insight into the chemical reactivities of the species.^44^ The increase in these values from analyte 1 to analyte 2 decreases the interaction of analyte 2 with the nonpolar binding pocket of the cyclodextrin hosts while the interactions between analyte 1 and the nonpolar binding pocket increase. Analytes 3 and 4 have similarly high polar surface areas relative to analyte 2, which explains why the analyte-induced modulation caused by analyte 1 is noticeably higher than those of the other analytes.

In contrast to analytes 2–4 which show markedly higher dipole moments and polar surface areas compared to analyte 1, analyte 5 has computed molecular properties that are remarkably similar to those of analyte 1. Nonetheless, the differences in how analytes 1 and 5 induce fluorescence modulation are substantial, with analyte 1 leading to markedly higher degrees of fluorescence modulation (*vide supra*). Such differences may be explained not by the Spartan computed structural information, but by the literature-reported photophysical activity of analyte 5,^28^ which can interfere with analyte-induced fluorescence modulation and complicate the observed results.

Finally, we note that qualitative kinetic analysis indicates that 48 hours were required for stabilization of the signal derived from Rhodamine 6G in the presence of the cyclodextrin hosts. We hypothesize that the reason for this long initial stabilization time derives from the time required to reach equilibrium of each rhodamine guest being bound in a corresponding cyclodextrin host. Of note, analogous equilibration times are well-precedented in the scientific literature.^45^

### Array generation experiments

**Fig. 5:**
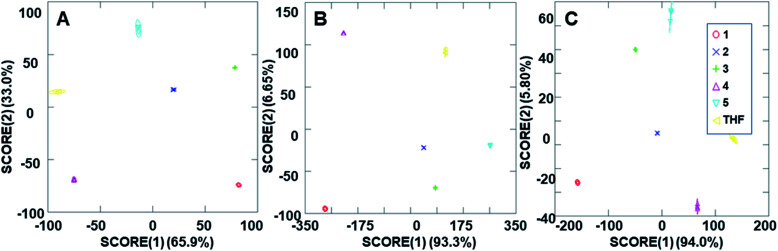
Arrays generated using the three cyclodextrin hosts as the predictors for the (A) 2 ppm trials, (B) 4 ppm trials and (C) 8 ppm trials.

The ability of the analyte-induced fluorescence modulation to provide unique, highly selective signals for each analyte was determined through the use of linear discriminant analysis (LDA). The use of the cyclodextrin hosts as predictors for the system enabled a well-separated signal for each analyte in the LDA plots, regardless of whether 5 μL, 10 μL, or 20 μL (6–30 μM) of the analyte solution was used (Fig. 5). The final concentrations of these analytes in the fluorescence modulation trials can be found in the ESI.† Of note, these plots include tetrahydrofuran (THF) as a control analyte, because that was the solvent used in the steroid solutions, and the well-separated signal of the THF solvent indicates that the analytes all induce fluorescence responses that are unique from the response generated by the solvent alone.

In addition to the well-separated signals between each analyte (apparent *via* visual inspection of Fig. 5), introduction of analytes as unknowns into the system after classification resulted in 100% accurate classification of the analytes (see ESI† for more details). Interestingly, as the additions increased in volume, the average cumulative proportion of total dispersion, a measure of the separation between the generated signals, also increased. Specifically, the 20 μL trials had an average of 96.7% dispersion, while the 5 μL and 10 μL trials had an average dispersion of 86.4% and 96.2% dispersion, respectively. This increase in dispersion at the highest analyte concentration is easily understandable, as higher concentrations of analytes lead to increased interactions with the fluorophore–cyclodextrin system and concomitant greater separation between signals.

**Fig. 6:**
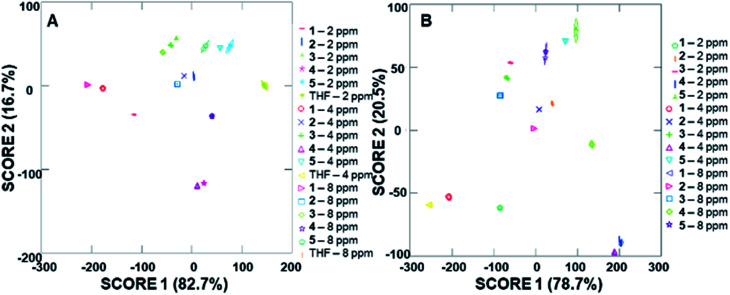
Arrays of fluorescence modulation data with three different addition volumes generated using the cyclodextrin hosts as predictors for all analytes (A) including THF as a control analyte; and (B) excluding THF as a control analyte.

Due to the differences in the observed cumulative proportions of total dispersion, we decided to further investigate system responsiveness to the analytes at the variety of concentrations investigated. Linear discriminant analysis of the signals generated from all analytes at all concentrations are summarized in Fig. 6A (with a THF control signal) and Fig. 6B (without the THF control signal included). Of note, although well-separated signals were generated for all analytes, the signal for THF in Fig. 6A has some overlap with the analytes investigated. When the THF signal was eliminated from the analysis, 100% separation was observed. Moreover, the closely grouped but still well-separated points on the plots represent the same analyte at different concentrations, which strongly suggests that quantitative analyte determination can also occur.

### Limit of detection and quantification experiments

In addition to determining the ability of the system to selectively distinguish between different analytes, the ability of the system to detect analytes at low concentrations is critically important in the development of practical detection devices. To that end, the limits of detection (defined as the lowest concentration of analyte that can be detected) and limits of quantification (defined as the lowest concentration of analyte that can be quantified) were calculated for all analytes in each of the cyclodextrin solutions, following literature reported procedures (see ESI† for more details), and key data is summarized in Table 4.

**Table 4:** Limits of detection (μM) calculated for analytes 1–5 in the cyclodextrin host systems^a^

Analyte	β-CD	Me-β-CD	2-HP-β-CD
1	17.0	5.30	2.34
2	11.4	3.89	3.35
3	8.91	0.148	1.89
4	7.67	5.98	0.775
5	6.11	0.049	3.59

a Values calculated using procedure found in the ESI. All results represent an average of at least four trials.

Overall, the limits of detection using unmodified β-cyclodextrin were higher than the limits of detection obtained using the other supramolecular hosts, and reflects both the lower modulation values as well as the presumed weaker interactions between β-cyclodextrin and the steroid analytes. Moreover, all limits of detection reported in Table 4 are markedly lower than the known concentrations of steroids found in urine testing following illegal doping activities,^46^ which highlights the potential of this system to be used in practical detection applications. We also note that although a variety of other interferents may be present in real-world systems, extensive previous work from our group has highlighted that cyclodextrin-based fluorescence modulation leads to highly analyte-specific responses, thus increasing the likelihood of being able to generate steroid-specific signals even in complex environments. Experiments to explicitly test the role of such interferents are currently underway in our laboratory.

## Conclusions

The fluorescence modulation system introduced herein as a sensor for anabolic steroids demonstrates significant promise as a tool for the detection of illegal doping. This system, which relies on cyclodextrin-promoted interactions between the target analytes and a high-quantum yield fluorophore, enables 100% selective differentiation between response patterns of the structurally similar anabolic steroids as well as different concentrations of the analytes, as well as limits of detection that were lower than concentrations reported in illegal doping scenarios. Future research in this area will focus on incorporating more analytes as well as on transitioning these promising results to a solid-state detection system. Work towards these goals is currently underway in our laboratory, and the results of these and other investigations will be reported in due course.

## Conflicts of interest

There are no conflicts of interest to declare.

## Supplementary Material

RA-010-D0RA03485A-s001
