# Supplementary material for: Detection of anabolic steroids via cyclodextrin-promoted fluorescence modulation
Source: RSC Adv. 2020 Jul 1;10(42):25108–15. doi: 10.1039/d0ra03485a (PMC9055181; doi:10.1039/d0ra03485a)
Supplement: RA-010-D0RA03485A-s001 [file RA-010-D0RA03485A-s001.pdf]

Electronic Supplementary Material (ESI) for RSC Advances.
This journal is © The Royal Society of Chemistry 2020

Electronic Supporting Information for

Detection of Anabolic Steroids via Cyclodextrin-Promoted Fluorescence Modulation

Anna Z. Haynes and Mindy Levine

Table of Contents

MATERIALS AND METHODS S3
DETAILS OF ANALYTES AND FLUOROPHORES S3
EXPERIMENTAL PROCEDURES S4
Experimental Procedure for Fluorescence Modulation Experiments S4
Experimental Procedure for Limit of Detection Experiments S5
Experimental Procedure for Array Generation Experiments S6
Experimental Procedure for Computational Experiments S7
SUMMARY TABLES S8
Summary Tables for Fluorescence Modulation Experiments S8
Summary Tables for Limit of Detection Experiments S9
Summary Tables for Array Generation Experiments S10
SUMMARY FIGURES S15
Summary Figures for Individual Fluorescence Modulation Experiments Using Fluorophore **6** S15
Summary Figures for Combined Fluorescence Modulation Experiments using Fluorophore **6** S35
Summary Figures for Limit of Detection (LOD) Experiments S37
Summary Figures for Array Generation Experiments S42
Summary Figures for Computational Experiments S47
REFERENCES S51

MATERIALS AND METHODS

The anabolic steroid analytes, chemicals required to make buffer solutions, fluorophore Rhodamine 6G, and solvent tetrahydrofuran were obtained from Sigma-Aldrich Chemical company and the cyclodextrins were obtained from Tokyo Chemical Industry (TCI). All chemicals were used as received without further purification. All fluorescence measurements were performed using a Shimadzu RF 6000 spectrophotometer. The excitation and emission slit widths were set to 3.0 nm. All fluorescence spectra were integrated vs. wavenumber on the X-axis using OriginPro 2019 Version 9.60. All arrays were generated using SYSTAT Version 13.1.

DETAILS OF ANALYTES AND FLUOROPHORES

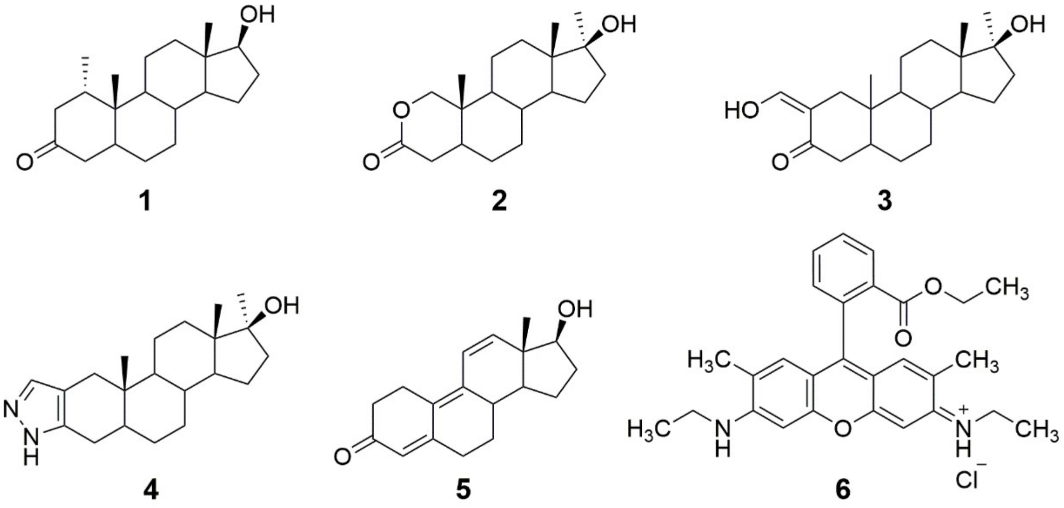

Figure S1: Structure of anabolic analytes (compound **1:** Mesterolone; compound **2**: Oxandrolone; compound **3**: Oxymetholone; compound **4**: Stanozolol; compound **5**: Trenbolone) and fluorophore Rhodamine 6G (compound **6**)

All analyte samples were prepared at a concentration of 1.0 mg/mL in THF. The fluorophore solution was prepared at a concentration of 0.1 mg/mL in THF. An 0.1 M citrate buffer was prepared by combining 2.409 grams of sodium citrate and 0.347 grams of citric acid in a 1.0 L volumetric flask and diluting to the mark with distilled water. The pH of the buffer was measured at 6.1, and remained consistent throughout the experimental procedures. Cyclodextrin solutions were prepared at a concentration of 10 mmol in the citrate buffer. The final concentrations of the analytes and fluorophore are shown in Table S1, below:

Table S1: Concentration of analytes and fluorophore in solution prior to dilution via sample preparation

Compound Number	Final Concentration (mM)
**1**	3.28
**2**	3.26
**3**	3.01
**4**	3.04
**5**	3.62
**6**	2.09

EXPERIMENTAL PROCEDURES

Experimental Procedure for Fluorescence Modulation Experiments

Fluorescence modulation experiments were done with 5 µL, 10 µL, and 20 µL sequential additions of analyte.

The fluorescence modulation values for each analyte-cyclodextrin combination were determined according to the following procedure:

1. 100 µL of fluorophore **6** solution (0.1 mg/mL) in THF was measured into six 15 mL glass vials (vial 1 for THF, vial 2 for analyte 1, vial 3 for analyte 2, etc.). 2.00 mL of a 10 mM cyclodextrin in citrate buffer and 0.40 mL 0.1 M citrate buffer were added to each (citrate buffer was at pH 6.1). The vials were capped and left to stabilize for 48 hours in a dark drawer.
2. After the 48 hours, the contents of one vial and 5.0 µL of analyte were added to the cuvette and stirred thoroughly to ensure homogeneity. The solution was excited at 490 nm and recorded from 500-800 nm. Four repeat measurements were taken. This step was repeated for each analyte and an additional time, adding 5.0 µL of THF instead of an analyte solution to use as a control.
3. Step 1 and 2 were repeated for each analyte-cyclodextrin combination (18 trials in total). In all cases, the solution was excited at the same wavelength (490 nm) and the emission spectra from 500 to 800 nm was recorded four times.
4. To conduct an experiment with no cyclodextrin present, step 1 was repeated but citrate buffer solution was substituted in place of the cyclodextrin solution. The final contents of the vials were then 100 µL of fluorophore **6** solution and 2.4 mL of 0.1 M citrate buffer.
5. After 48 hours, step 2 was repeated using this set of solutions containing no cyclodextrin for each analyte. The solutions were excited at the same wavelength (490 nm) and the emission spectra from 500 to 800 nm was recorded four times.
6. Emission spectra were integrated versus wavenumber on the X-axis using OriginPro software and fluorescence modulation ratios were determined according to Equation 1, below:

   $$\text{Fluorophore Modulation Ratios} = Fl_{\text{analyte}} / Fl_{\text{blank}} \qquad \text{(Eq. S1)}$$

   where Fl_{analyte} represents the integrated fluorescence emission of the fluorophore in the presence of the analyte and Fl_{blank} represents the integrated fluorescence emission of the fluorophore in the absence of the analyte.
7. These ratios were recordedEsi .

Step 2 and 5 were repeated with sequential additions for total addition amounts of 10 µL and 20 µL of analyte. The final concentrations of the analytes and the fluorophore in solution using each of the three addition protocols are summarized in Table S2, below:

Table S2: Final concentrations (µM) of analytes and fluorophore in fluorescence modulation trials

Compound	5 µL	10 µL	20 µL
**1**	6.419	12.81	25.53
**2**	6.419	12.81	25.53
**3**	5.890	11.76	23.42
**4**	5.949	11.88	23.67
**5**	7.226	14.42	28.73
**6**	8.334	8.317	8.284

Experimental Procedure for Limit of Detection Experiments

The limit of detection (LOD), defined as the lowest concentration of the analyte that can be detected, was obtained using the calibration curve method, following procedures reported by Loock et. al.[1] The limit of quantification (LOQ) is the lowest concentration of analyte that can be reliably and accurately quantified. The limit of detection and quantification experiments were conducted following literature-reported procedures.[1,2]

LOD experiments were done with sequential 5 µL additions of analyte, according to the procedures listed below:

1. 100 µL of fluorophore **6** solution (0.1 mg/mL) in THF was measured into a 15 mL glass vial. 2.00 mL of a 10 mM cyclodextrin in citrate buffer and 0.40 mL 0.1 M citrate buffer were added (citrate buffer was at pH 6). The vial was capped and left to stabilize for 48 hours.
2. The solution was transferred to a quartz cuvette and then excited at 490 nm and the fluorescence emission spectra was recorded from 500 to 800 nm. Each fluorescence measurement was repeated six times
3. 5.0 µL of the analyte solution in THF was added to the cuvette and stirred thoroughly to ensure homogeneity. The solution was excited at the same wavelength (490 nm) and the emission was measured between 500 nm and 800 nm. Six repeat measurements were taken.
4. Step 2 was repeated four times for total addition volumes of 10 µL, 15 µL, 20 µL, and 25 µL of the analyte solution. In all cases, the solution was excited at the same wavelength (490 nm) and the emission spectra from 500 to 800 nm was recorded six times.
5. Emission spectra were integrated versus wavenumber on the X-axis using OriginPro software, and were used to generate calibration curves with analyte concentration on the X-axis and integrated fluorescence emission on the Y-axis. The curve was fitted with a linear trendline and the equation of the line was determined.
6. The measurements from Step 1, the emission spectra of the combination of the Rhodamine solution and β-cyclodextrin solution with no addition of analyte, are referred to as the blank in the following calculations.
7. The limit of the blank (LOD_{blank}) is defined according to the following equation:

$$LOD_{blank} = m_{blank} - 3(SD_{blank}) \quad \text{(Eq.S2)}$$

   where m_{blank} is the mean of the blank integrations and SD_{blank} is the standard deviation of those measurements.
8. The LOD_{blank} was then entered into the equation determined in Step 4 as the y-value. The corresponding x-value was calculated. This value is the LOD of the analyte in µM in the system.
9. The LOQ (LOQ_{blank}) was calculated in a similar manner to the LOD. The limit of quantification of the blank is defined according to the following equation:

$$LOQ_{blank} = m_{blank} - 10(SD_{blank}). \quad \text{(Eq. S3)}$$

   This value is then entered as the y-value from step 4 and the corresponding x-value was calculated. This is the value of the LOQ of the analyte for the system in µM.

All steps were repeated for each analyte-cyclodextrin combination (5 analytes x 3 cyclodextrin solutions = 15 trials).

The summary tables of these results for each analyte-cyclodextrin combination are shown in Tables S4-S6 (*vide infra*).

Experimental Procedure for Array Generation Experiments

Linear discriminant analysis was performed using SYSTAT 13 statistical computing software with the following software settings[3]:

(a) Linear Discriminant Analysis

(b) Grouping Variable: Analytes

(c) Predictors: Cyclodextrin hosts

(d) Long-Range Statistics: Mahal.

These experiments were then repeated using only two predictors (i.e. cyclodextrins) instead of all three, and the results of array-based analysis for each pair of predictors is reported herein as well.

Experimental Procedure for Computational Experiments

Spartan software version '18 was used to calculate the equilibrium molecular conformations of each analyte in their ground states in the gas phase using a semi-empirical PM3 model for each analyte. This allowed an electrostatic potential map surface to be overlaid over the molecules, using the mesh overlay function.

SUMMARY TABLES

Summary Tables for Fluorescence Modulation Experiments

Table S3. Fluorescence modulation results obtained for analytes **1-5** with various cyclodextrins in the presence of fluorophore **6**[a]

Addition amount	Analyte	β-Cyclodextrin (β-CD)	Methyl-β-Cyclodextrin (Me-β-CD)	2-Hydroxypropyl-β-Cyclodextrin (2-HPCD)	No Cyclodextrin (CD)
5 μL	**1**	0.958 ± 0.001	0.956 ± 0.000	0.905 ± 0.000	1.001 ± 0.001
	**2**	0.997 ± 0.001	0.971 ± 0.000	0.957 ± 0.000	0.973 ± 0.001
	**3**	1.004 ± 0.001	0.956 ± 0.000	0.966 ± 0.000	1.006 ± 0.001
	**4**	0.989 ± 0.003	0.998 ± 0.001	0.906 ± 0.000	0.979 ± 0.001
	**5**	0.997 ± 0.001	0.978 ± 0.001	0.999 ± 0.001	1.047 ± 0.001
10 μL	**1**	0.950 ± 0.000	0.938 ± 0.000	0.900 ± 0.000	1.004 ± 0.001
	**2**	0.990 ± 0.001	0.967 ± 0.000	0.953 ± 0.000	0.990 ± 0.000
	**3**	0.988 ± 0.001	0.955 ± 0.000	0.962 ± 0.000	1.009 ± 0.001
	**4**	0.985 ± 0.002	0.995 ± 0.000	0.901 ± 0.000	0.969 ± 0.002
	**5**	0.992 ± 0.001	0.975 ± 0.001	0.992 ± 0.000	1.040 ± 0.000
20 μL	**1**	0.948 ± 0.001	0.932 ± 0.000	0.892 ± 0.000	0.994 ± 0.001
	**2**	0.974 ± 0.001	0.966 ± 0.001	0.947 ± 0.000	0.987 ± 0.000
	**3**	0.978 ± 0.001	0.954 ± 0.000	0.955 ± 0.000	1.004 ± 0.003
	**4**	0.975 ± 0.000	0.986 ± 0.001	0.952 ± 0.000	0.972 ± 0.001
	**5**	0.975 ± 0.001	0.969 ± 0.001	0.988 ± 0.001	1.036 ± 0.001

[a] All values were calculated using Equation S1, and results reported represent an average of at least four trials.

Summary Tables for Limit of Detection Experiments

Table S4. Summary table for limits of detection experiments with β-CD[a,b]

Analyte	LOD (μM)	LOQ (μM)	Equation	R^2
**1**	17.03	46.58	y = -1292.6693x + 2133565.2492	0.8369
**2**	11.39	35.32	y = -2079.0320x + 2229038.8629	0.9589
**3**	8.914	21.42	y = -2559.6708x + 2233461.8386	0.9308
**4**	7.665	30.49	y = -2237.8468x + 2220576.1935	0.9776
**5**	6.108	24.35	y = -2308.8616x + 2241047.0782	0.9498

[a] All LOD values were calculated using Equation S2.
[a] All LOQ values were calculated using Equation S3.

Table S5. Summary table for limits of detection experiments with Me-β-CD[a,b]

Analyte	LOD (μM)	LOQ (μM)	Equation	R^2
**1**	5.300	11.72	y = -27374.1167x + 31229827.0035	0.8451
**2**	3.886	18.34	y = -11127.1906x + 31855034.7259	0.9193
**3**	0.148	4.102	y = -9482.3445x + 31383949.2786	0.8929
**4**	5.981	18.21	y = -16056.2718x + 32744010.5956	0.9379
**5**	0.049	4.586	y = -21626.3876x + 32220132.7800	0.9755

[a] All LOD values were calculated using Equation S2.
[a] All LOQ values were calculated using Equation S3.

Table S6. Summary table for limits of detection experiments with 2-HPCD[a,b]

Analyte	LOD (μM)	LOQ (μM)	Equation	R^2
**1**	2.335	7.129	y = -18245.1311x + 26534054.7777	0.9956
**2**	3.352	14.66	y = -12790.4792x + 28037979.4349	0.9657
**3**	1.886	8.361	y = -21071.8113x + 28366093.4431	0.9945
**4**	0.775	3.371	y = -10283.4719x + 26474157.9172	0.8972
**5**	3.587	11.53	y = -11788.1436x + 29202530.0773	0.9438

[a] All LOD values were calculated using Equation S2.
[a] All LOQ values were calculated using Equation S3.

Summary Tables for Array Generation Experiments

With 5 µL analyte additions

Table S7. Linear discriminant analysis results with β-CD, Me-β-CD, and 2-HPCD as predictors

Jackknifed Classification Matrix

	1	2	3	4	5	THF	%correct
1	4	0	0	0	0	0	100
2	0	4	0	0	0	0	100
3	0	0	4	0	0	0	100
4	0	0	0	4	0	0	100
5	0	0	0	0	4	0	100
THF	0	0	0	0	0	4	100
Total	4	4	4	4	4	4	100

Cumulative Proportion of Total Dispersion

0.601	0.990	1.000

Table S8. Linear discriminant analysis results using β-CD and Me-β-CD as predictors

Jackknifed Classification Matrix

	1	2	3	4	5	THF	%correct
1	4	0	0	0	0	0	100
2	0	4	0	0	0	0	100
3	0	0	4	0	0	0	100
4	0	0	0	4	0	0	100
5	0	0	0	0	4	0	100
THF	0	0	0	0	0	4	100
Total	4	4	4	4	4	4	100

Cumulative Proportion of Total Dispersion

0.951	1.000

Table S9. Linear discriminant analysis results using β-CD and 2-HPCD as predictors

Jackknifed Classification Matrix

	1	2	3	4	5	THF	%correct
1	4	0	0	0	0	0	100
2	0	4	0	0	0	0	100
3	0	0	4	0	0	0	100
4	0	0	0	4	0	0	100
5	0	0	0	0	4	0	100
THF	0	0	0	0	1	3	75
Total	4	4	4	4	5	3	96

Cumulative Proportion of Total Dispersion

0.973	1.000

Table S10. Linear discriminant analysis results using Me-β-CD and 2-HPCD as predictors

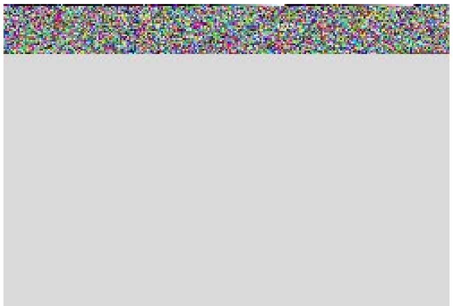

Cumulative Proportion of Total Dispersion

0.711	1.000

With 10 μL analyte additions

Table S11. Linear discriminant analysis results with β-CD, Me-β-CD, and 2-HPCD as predictors

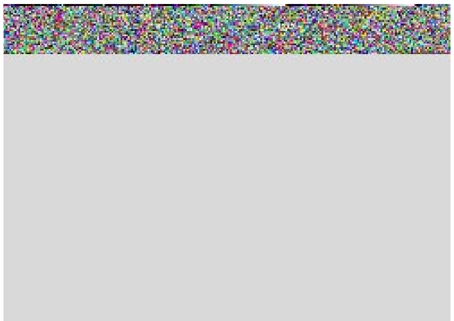

Cumulative Proportion of Total Dispersion

0.888	0.999	1.000

Table S12. Linear discriminant analysis results using β-CD and Me-β-CD as predictors

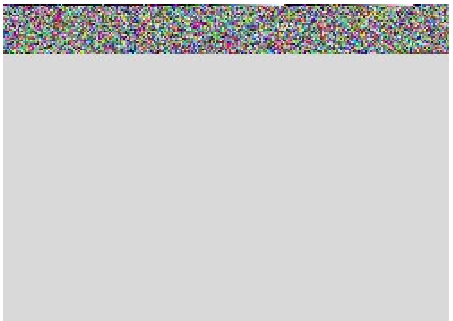

Cumulative Proportion of Total Dispersion

0.981	1.000

Table S13. Linear discriminant analysis results using β-CD and 2-HPCD as predictors

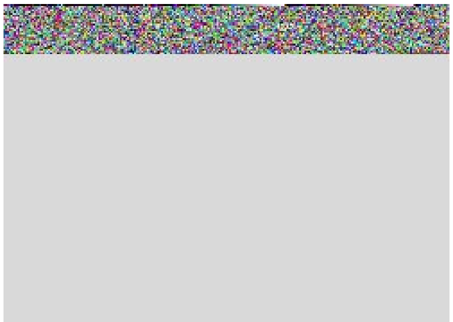

Cumulative Proportion of Total Dispersion

0.997	1.000

Table S14. Linear discriminant analysis results using Me-β-CD and 2-HPCD as predictors

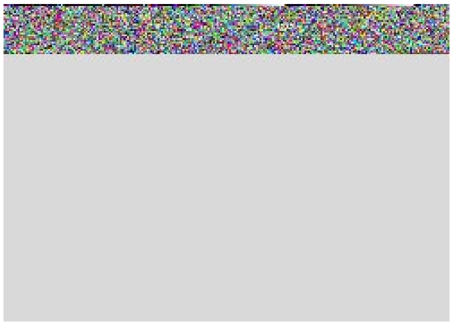

Cumulative Proportion of Total Dispersion

0.916	1.000

<u>With 20 µL analyte additions</u>

Table S15. Linear discriminant analysis results using β-CD, Me-β-CD, and 2-HPCD as predictors

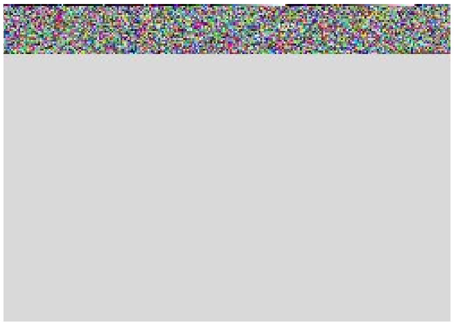

Cumulative Proportion of Total Dispersion

0.906	0.996	1.000

Table S16. Linear discriminant analysis results using β-CD and Me-β-CD as predictors

[illegible]

Cumulative Proportion of Total Dispersion	
0.993	1.000

Table S17. Linear discriminant analysis results using β-CD and 2-HPCD as predictors

[illegible]

Cumulative Proportion of Total Dispersion	
0.988	1.000

Table S18. Linear discriminant analysis results using Me-β-CD and 2-HPCD as predictors

[illegible]

Cumulative Proportion of Total Dispersion	
0.944	1.000

All Additions with THF

Table S19. Linear discriminant analysis results with β-CD, Me-β-CD, and 2-HPCD as predictors

Jackknifed Classification Matrix

	10uL - 1	10uL - 2	10uL - 3	10uL - 4	10uL - 5	10uL - THF	20uL - 1	20uL - 2	20uL - 3	20uL - 4	20uL - 5	20uL - THF	5uL - 1	5uL - 2	5uL - 3	5uL - 4	5uL - 5	5uL - THF	%correct
10uL - 1	4	0	0	0	0	0	0	0	0	0	0	0	0	0	0	0	0	0	100
10uL - 2	0	4	0	0	0	0	0	0	0	0	0	0	0	0	0	0	0	0	100
10uL - 3	0	0	4	0	0	0	0	0	0	0	0	0	0	0	0	0	0	0	100
10uL - 4	0	0	0	4	0	0	0	0	0	0	0	0	0	0	0	0	0	0	100
10uL - 5	0	0	0	0	4	0	0	0	0	0	0	0	0	0	0	0	0	0	100
10uL - THF	0	0	0	0	0	0	0	0	0	0	0	4	0	0	0	0	0	0	0
20uL - 1	0	0	0	0	0	0	4	0	0	0	0	0	0	0	0	0	0	0	100
20uL - 2	0	0	0	0	0	0	0	4	0	0	0	0	0	0	0	0	0	0	100
20uL - 3	0	0	0	0	0	0	0	0	4	0	0	0	0	0	0	0	0	0	100
20uL - 4	0	0	0	0	0	0	0	0	0	4	0	0	0	0	0	0	0	0	100
20uL - 5	0	0	0	0	0	0	0	0	0	0	4	0	0	0	0	0	0	0	100
20uL - THF	0	0	0	0	0	4	0	0	0	0	0	0	0	0	0	0	0	0	0
5uL - 1	0	0	0	0	0	0	0	0	0	0	0	0	4	0	0	0	0	0	100
5uL - 2	0	0	0	0	0	0	0	0	0	0	0	0	0	4	0	0	0	0	100
5uL - 3	0	0	0	0	0	0	0	0	0	0	0	0	0	0	4	0	0	0	100
5uL - 4	0	0	0	0	0	0	0	0	0	0	0	0	0	0	0	4	0	0	100
5uL - 5	0	0	0	0	0	0	0	0	0	0	0	0	0	0	0	0	4	0	100
5uL - THF	0	0	0	0	0	4	0	0	0	0	0	0	0	0	0	0	0	0	0
Total	4	4	4	4	4	8	4	4	4	4	4	4	4	4	4	4	4	0	83

Cumulative Proportion of Total Dispersion

0.787	0.992	1.000

All Additions excluding THF

Table S20. Linear discriminant analysis results with β-CD, Me-β-CD, and 2-HPCD as predictors

Jackknifed Classification Matrix

	10uL - 1	10uL - 2	10uL - 3	10uL - 4	10uL - 5	20uL - 1	20uL - 2	20uL - 3	20uL - 4	20uL - 5	5uL - 1	5uL - 2	5uL - 3	5uL - 4	5uL - 5	%correct
10uL - 1	4	0	0	0	0	0	0	0	0	0	0	0	0	0	0	100
10uL - 2	0	4	0	0	0	0	0	0	0	0	0	0	0	0	0	100
10uL - 3	0	0	4	0	0	0	0	0	0	0	0	0	0	0	0	100
10uL - 4	0	0	0	4	0	0	0	0	0	0	0	0	0	0	0	100
10uL - 5	0	0	0	0	4	0	0	0	0	0	0	0	0	0	0	100
20uL - 1	0	0	0	0	0	4	0	0	0	0	0	0	0	0	0	100
20uL - 2	0	0	0	0	0	0	4	0	0	0	0	0	0	0	0	100
20uL - 3	0	0	0	0	0	0	0	4	0	0	0	0	0	0	0	100
20uL - 4	0	0	0	0	0	0	0	0	4	0	0	0	0	0	0	100
20uL - 5	0	0	0	0	0	0	0	0	0	4	0	0	0	0	0	100
5uL - 1	0	0	0	0	0	0	0	0	0	0	4	0	0	0	0	100
5uL - 2	0	0	0	0	0	0	0	0	0	0	0	4	0	0	0	100
5uL - 3	0	0	0	0	0	0	0	0	0	0	0	0	4	0	0	100
5uL - 4	0	0	0	0	0	0	0	0	0	0	0	0	0	4	0	100
5uL - 5	0	0	0	0	0	0	0	0	0	0	0	0	0	0	4	100
Total	4	4	4	4	4	4	4	4	4	4	4	4	4	4	4	100

Cumulative Proportion of Total Dispersion

0.827	0.993	1.000

SUMMARY FIGURES

Summary Figures for Individual Fluorescence Modulation Experiments Using Fluorophore 6

With 5 μL analyte addition

β-CD

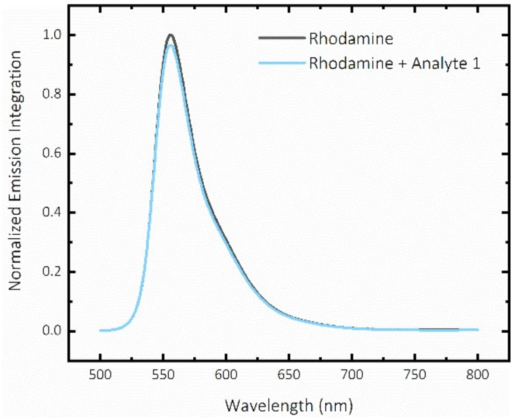

Figure S2. Fluorescence modulation of analyte **1** in the presence of β-CD

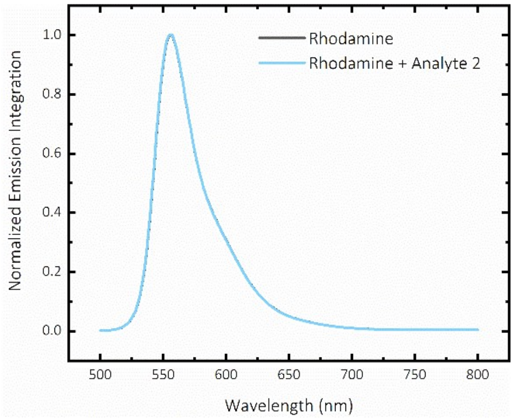

Figure S3. Fluorescence modulation of analyte **2** in the presence of β-CD

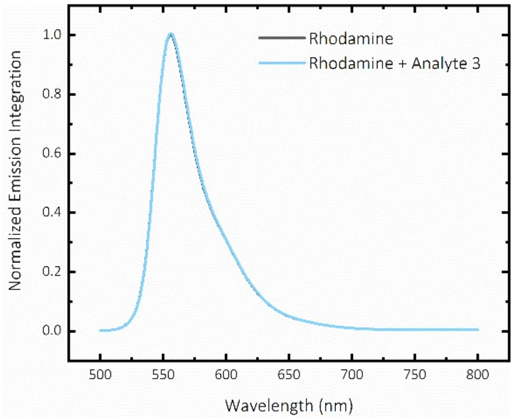

Figure S4. Fluorescence modulation of analyte **3** in the presence of β-CD

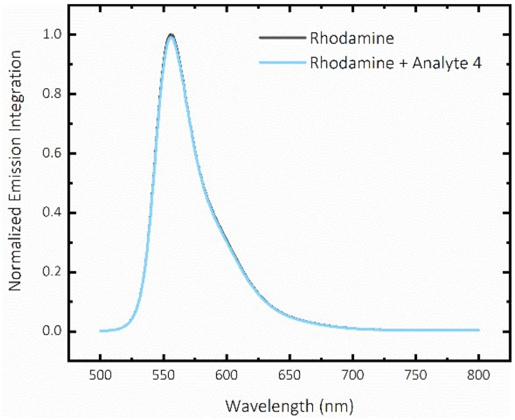

Figure S5. Fluorescence modulation of analyte **4** in the presence of β-CD

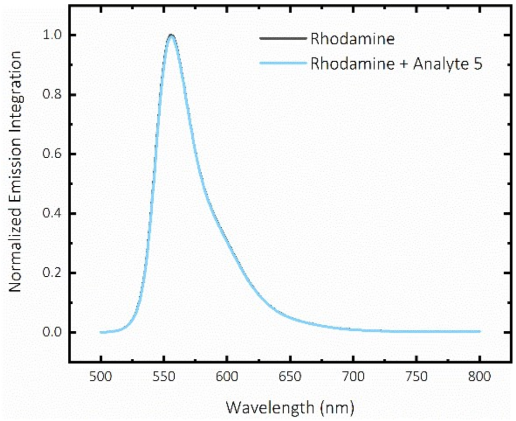

Figure S6. Fluorescence modulation of analyte **5** in the presence of β-CD

Me-β-CD

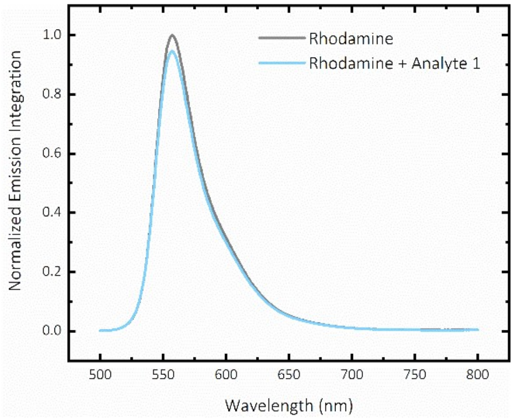

Figure S7. Fluorescence modulation of analyte **1** in the presence of Me-β-CD

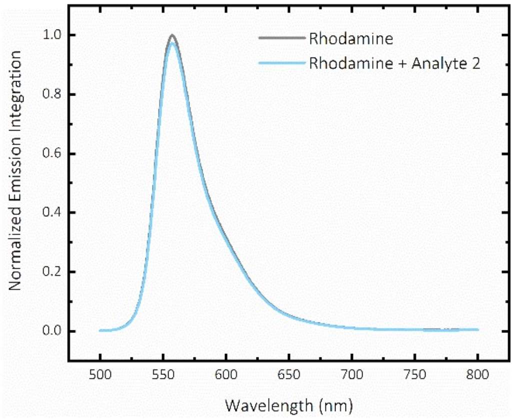

Figure S8. Fluorescence modulation of analyte **2** in the presence of Me-β-CD

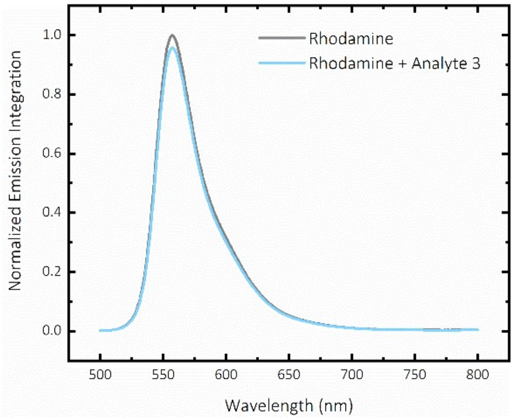

Figure S9. Fluorescence modulation of analyte **3** in the presence of Me-β-CD

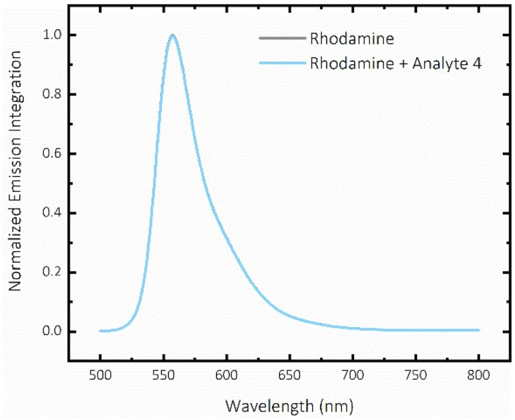

Figure S10. Fluorescence modulation of analyte **4** in the presence of Me-β-CD

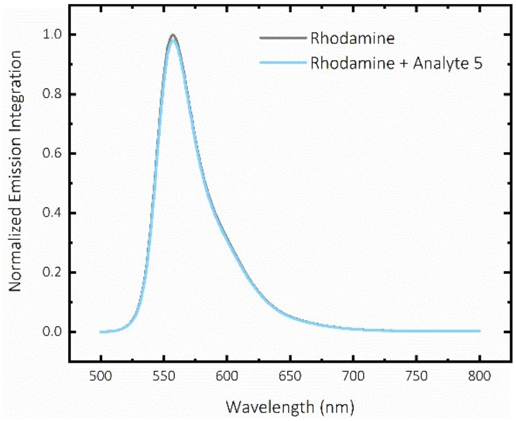

Figure S11. Fluorescence modulation of analyte **5** in the presence of Me-β-CD

2-HPCD

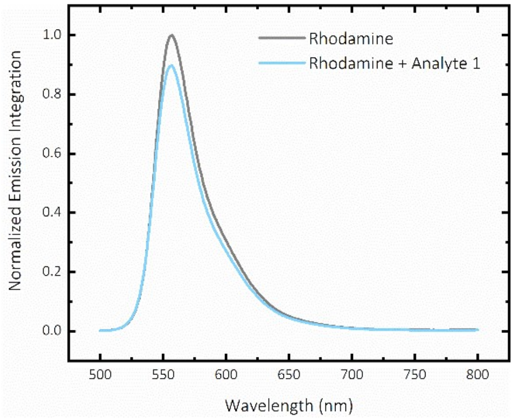

Figure S12. Fluorescence modulation of analyte **1** in the presence of 2-HPCD

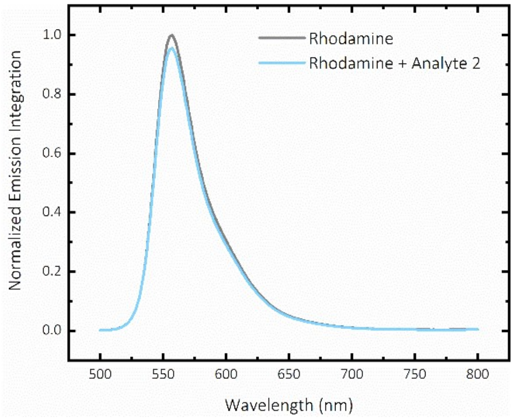

Figure S13. Fluorescence modulation of analyte **2** in the presence of 2-HPCD

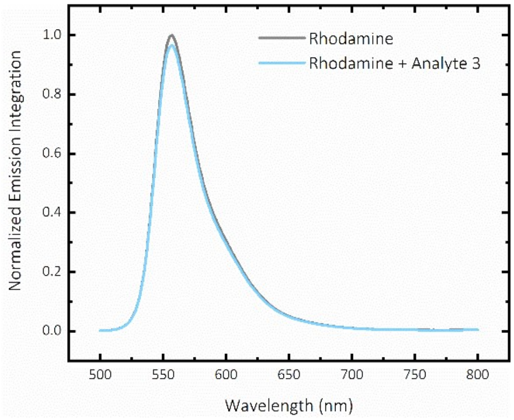

Figure S14. Fluorescence modulation of analyte **3** in the presence of 2-HPCD

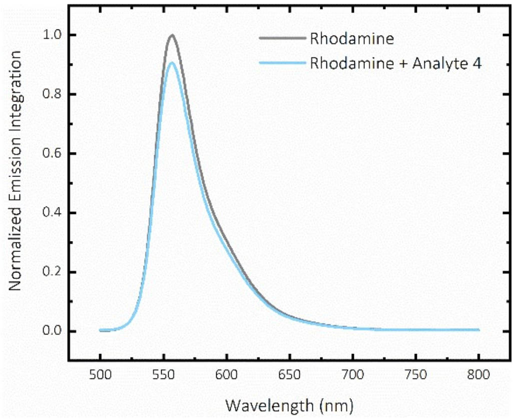

Figure S15. Fluorescence modulation of analyte **4** in the presence of 2-HPCD

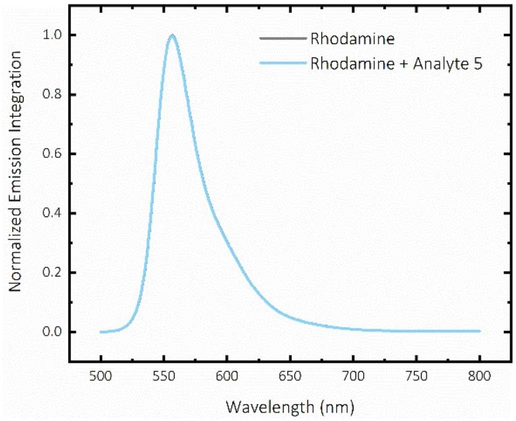

Figure S16. Fluorescence modulation of analyte **5** in the presence of 2-HPCD

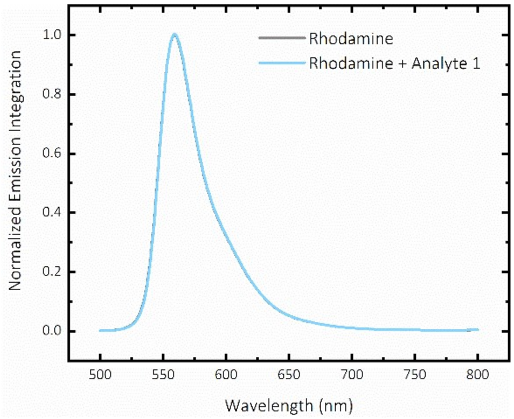

Figure S17. Fluorescence modulation of analyte **1** in the presence of no CD

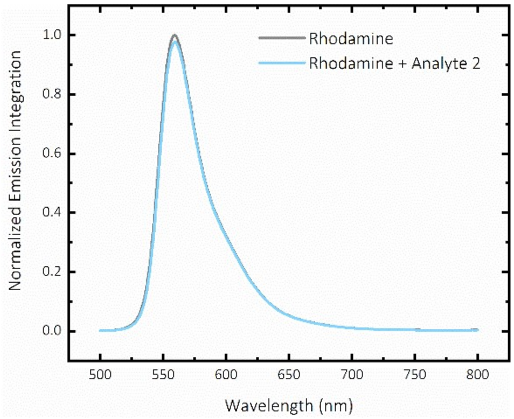

Figure S18. Fluorescence modulation of analyte **2** in the presence of no CD

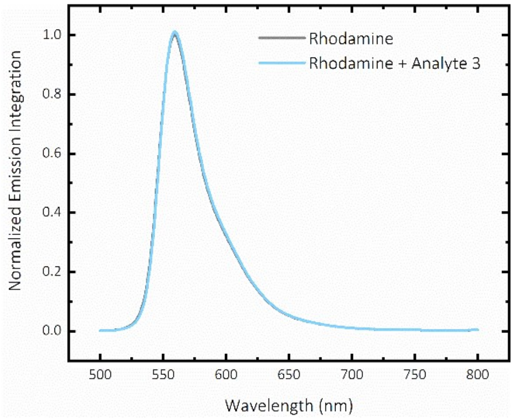

Figure S19. Fluorescence modulation of analyte **3** in the presence of no CD

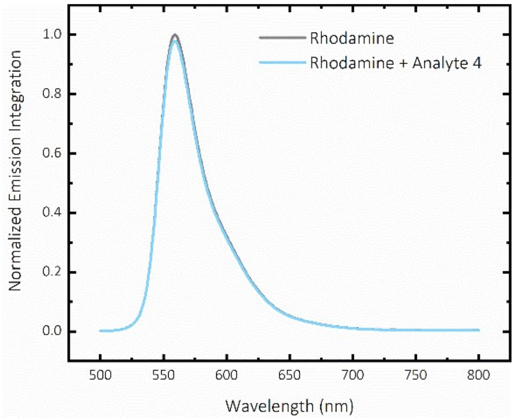

Figure S20. Fluorescence modulation of analyte **4** in the presence of no CD

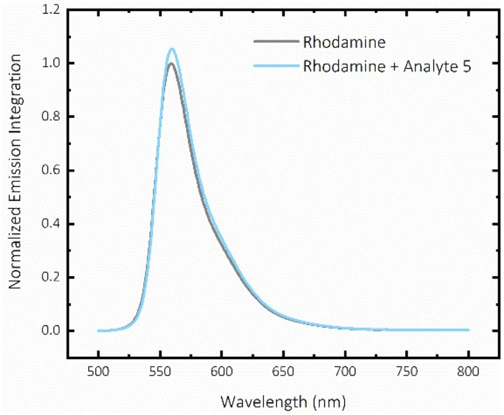

Figure S21. Fluorescence modulation of analyte **5** in the presence of no CD

<u>With 10 μL analyte addition</u>

<u>β-CD</u>

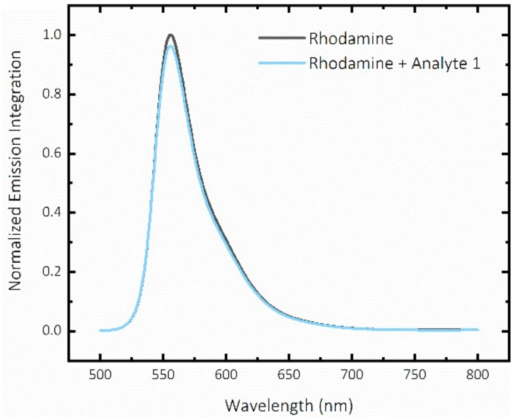

Figure S22. Fluorescence modulation of analyte **1** in the presence of β-CD

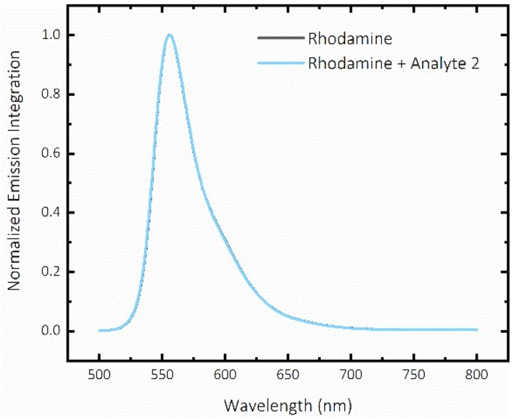

Figure S23. Fluorescence modulation of analyte **2** in the presence of β-CD

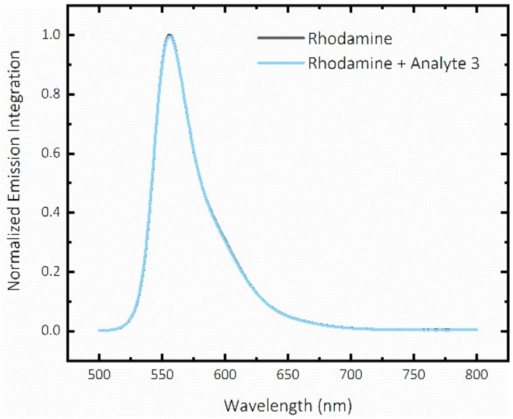

Figure S24. Fluorescence modulation of analyte **3** in the presence of β-CD

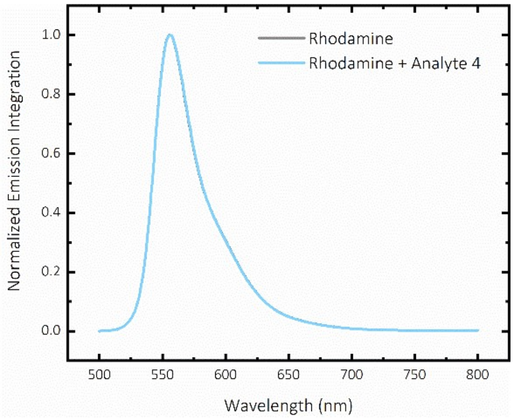

Figure S25. Fluorescence modulation of analyte **4** in the presence of β-CD

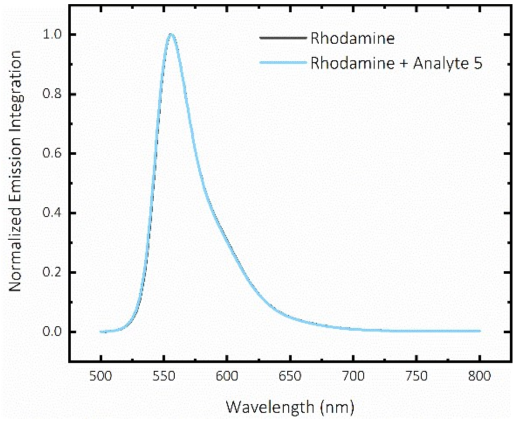

Figure S26. Fluorescence modulation of analyte **5** in the presence of β-CD

Me-β-CD

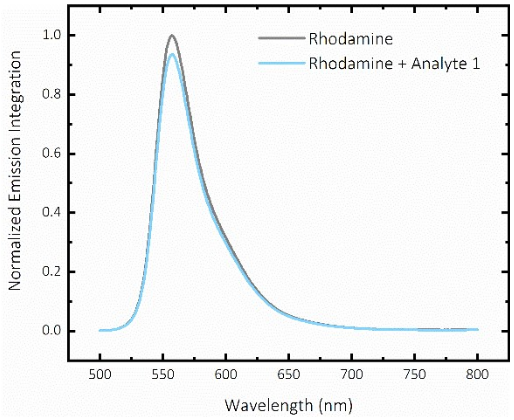

Figure S27. Fluorescence modulation of analyte **1** in the presence of Me-β-CD

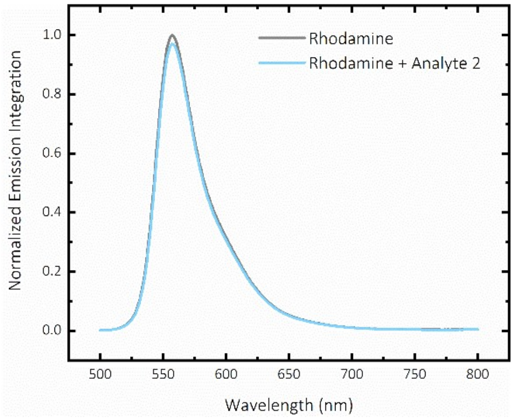

Figure S28. Fluorescence modulation of analyte **2** in the presence of Me-β-CD

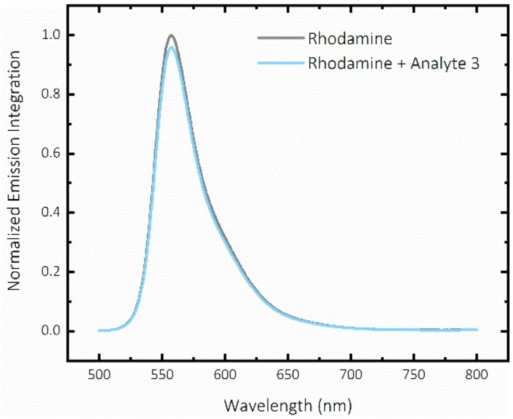

Figure S29. Fluorescence modulation of analyte **3** in the presence of Me-β-CD

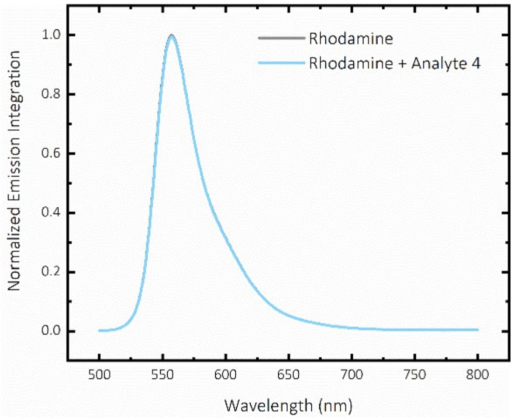

Figure S30. Fluorescence modulation of analyte **4** in the presence of Me-β-CD

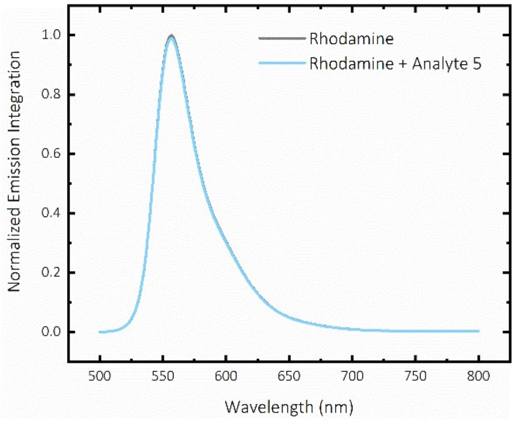

Figure S31. Fluorescence modulation of analyte **5** in the presence of Me-β-CD

2-HPCD

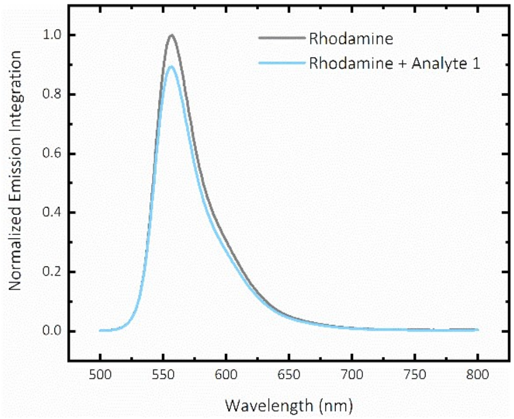

Figure S32. Fluorescence modulation of analyte **1** in the presence of 2-HPCD

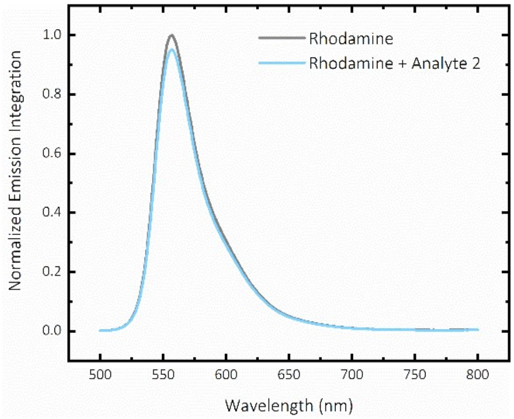

Figure S33. Fluorescence modulation of analyte **2** in the presence of 2-HPCD

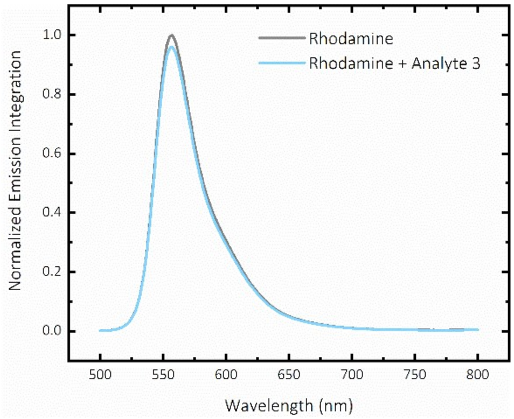

Figure S34. Fluorescence modulation of analyte **3** in the presence of 2-HPCD

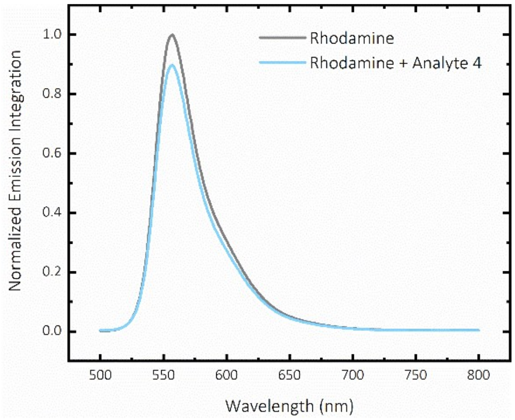

Figure S35. Fluorescence modulation of analyte **4** in the presence of 2-HPCD

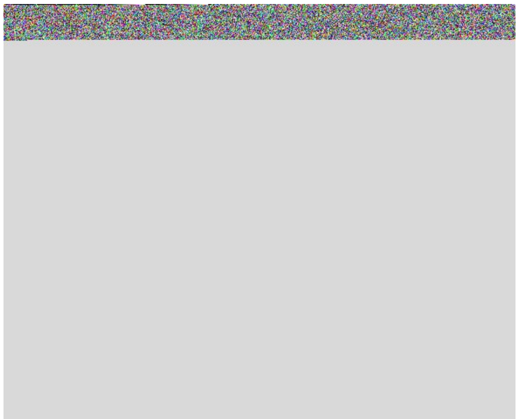

Figure S36. Fluorescence modulation of analyte **5** in the presence of 2-HPCD

No CD

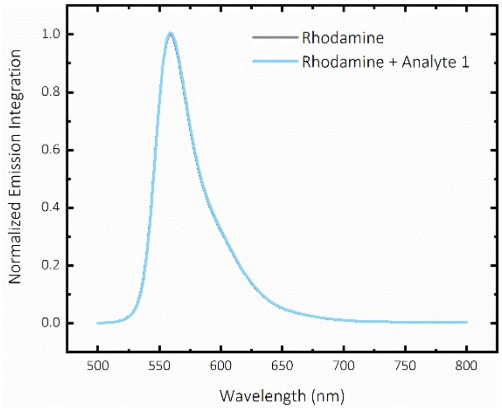

Figure S37. Fluorescence modulation of analyte **1** in the presence of no CD

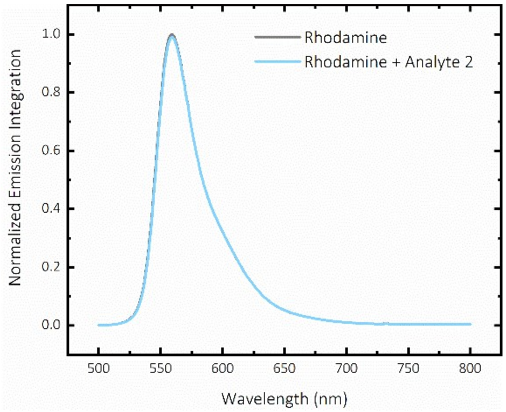

Figure S38. Fluorescence modulation of analyte **2** in the presence of no CD

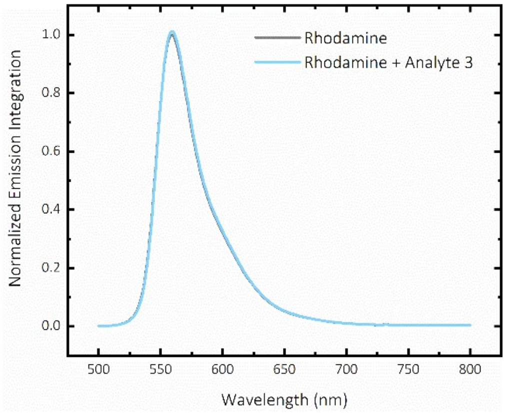

Figure S39. Fluorescence modulation of analyte **3** in the presence of no CD

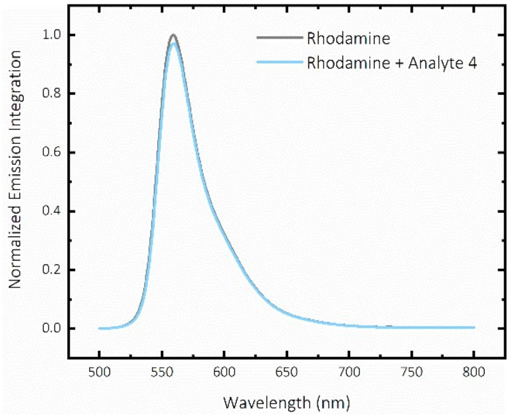

Figure S40. Fluorescence modulation of analyte **4** in the presence of no CD

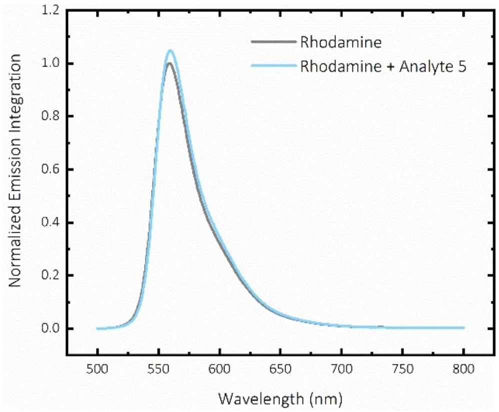

Figure S41. Fluorescence modulation of analyte **5** in the presence of no CD

With 20 μL analyte addition

β-CD

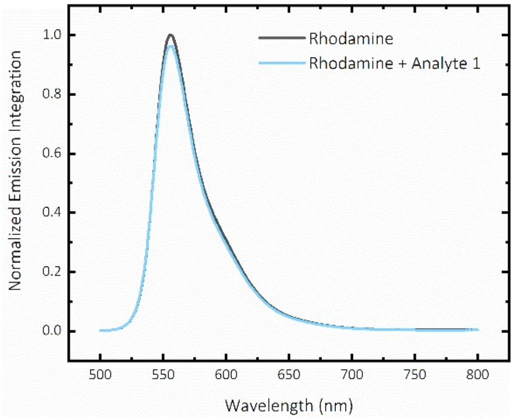

Figure S42. Fluorescence modulation of analyte **1** in the presence of β-CD

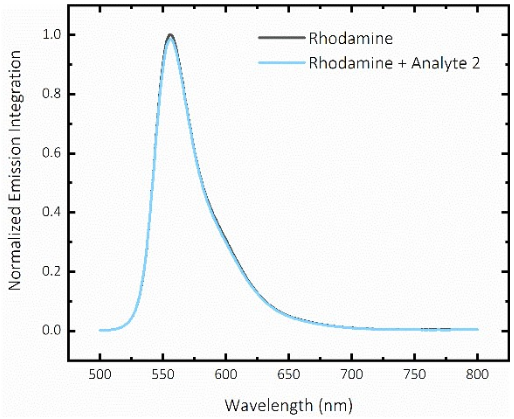

Figure S43. Fluorescence modulation of analyte **2** in the presence of β-CD

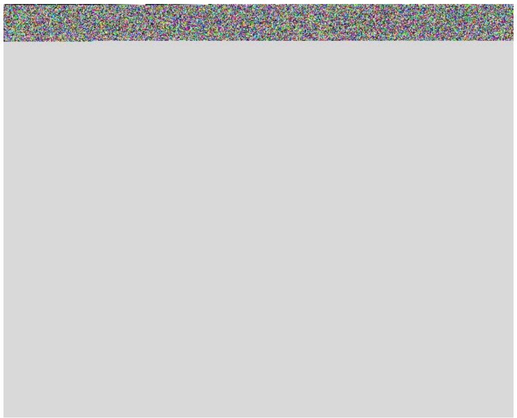

Figure S44. Fluorescence modulation of analyte **3** in the presence of β-CD

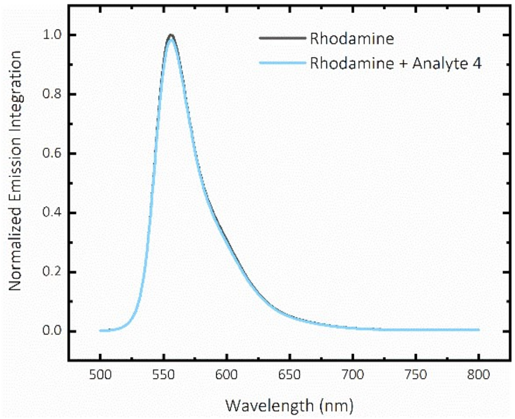

Figure S45. Fluorescence modulation of analyte **4** in the presence of β-CD

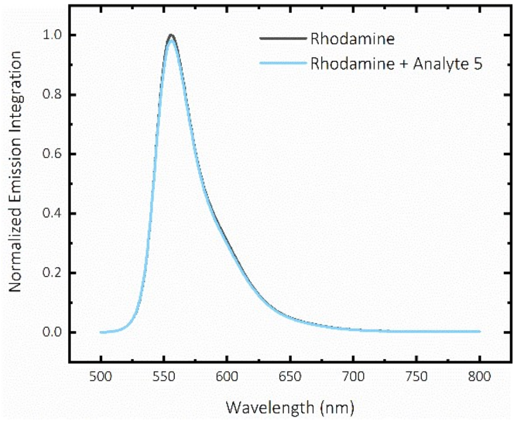

Figure S46. Fluorescence modulation of analyte **5** in the presence of β-CD

Me-β-CD

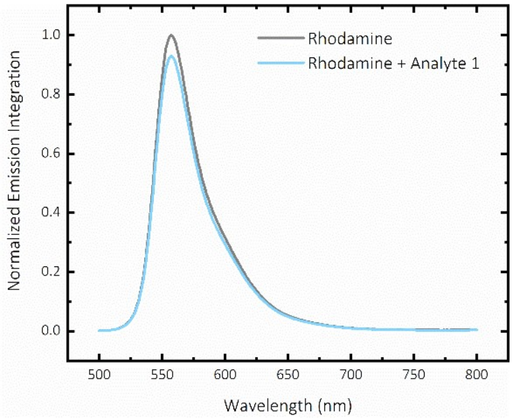

Figure S47. Fluorescence modulation of analyte **1** in the presence of Me-β-CD

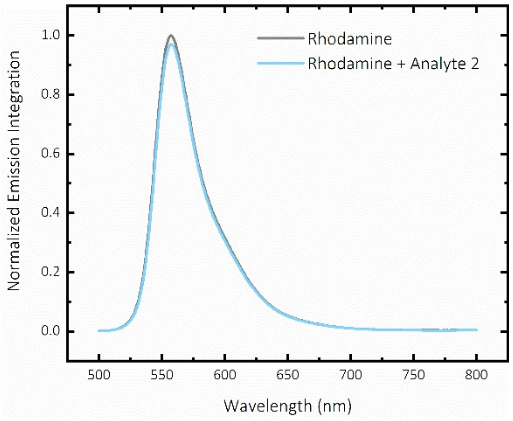

Figure S48. Fluorescence modulation of analyte **2** in the presence of Me-β-CD

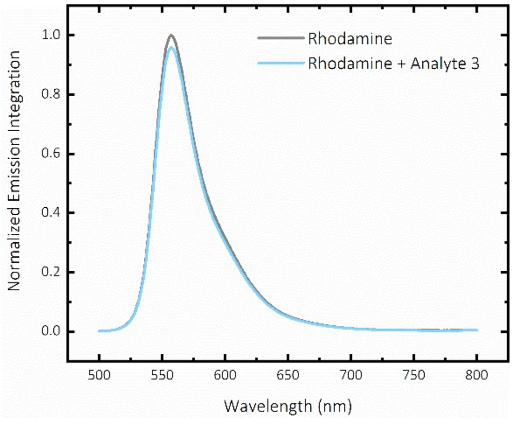

Figure S49. Fluorescence modulation of analyte **3** in the presence of Me-β-CD

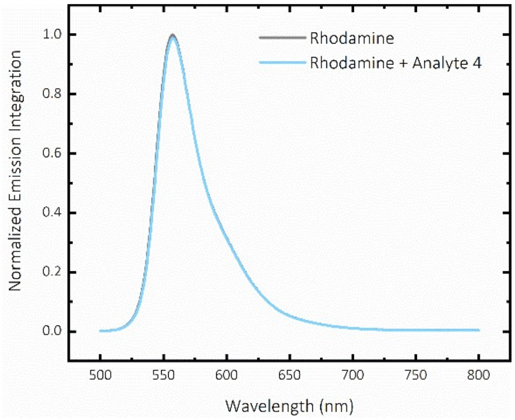

Figure S50. Fluorescence modulation of analyte **4** in the presence of Me-β-CD

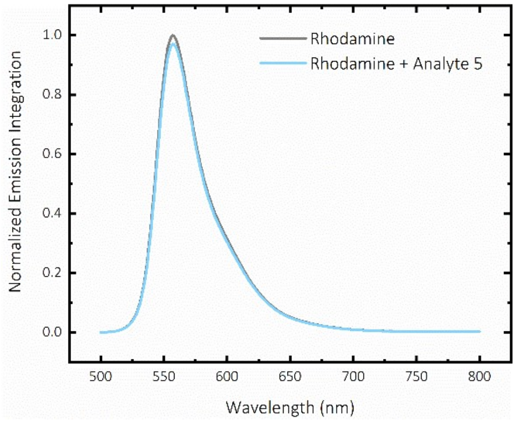

Figure S51. Fluorescence modulation of analyte **5** in the presence of Me-β-CD

2-HPCD

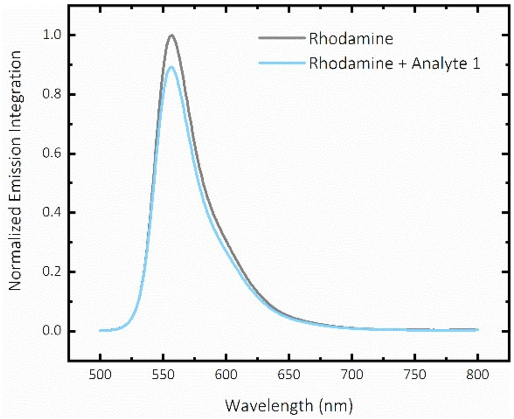

Figure S52. Fluorescence modulation of analyte **1** in the presence of 2-HPCD

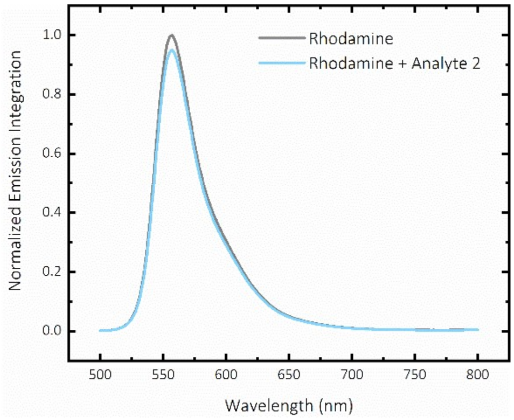

Figure S53. Fluorescence modulation of analyte **2** in the presence of 2-HPCD

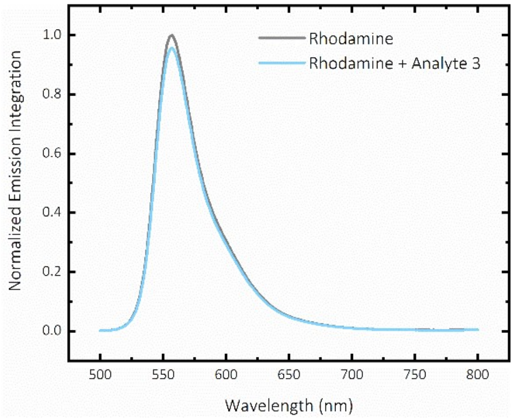

Figure S54. Fluorescence modulation of analyte **3** in the presence of 2-HPCD

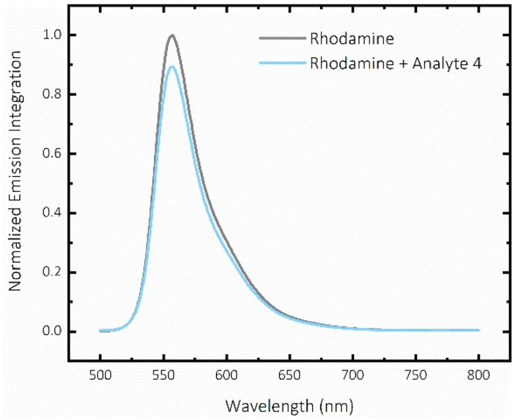

Figure S55. Fluorescence modulation of analyte **4** in the presence of 2-HPCD

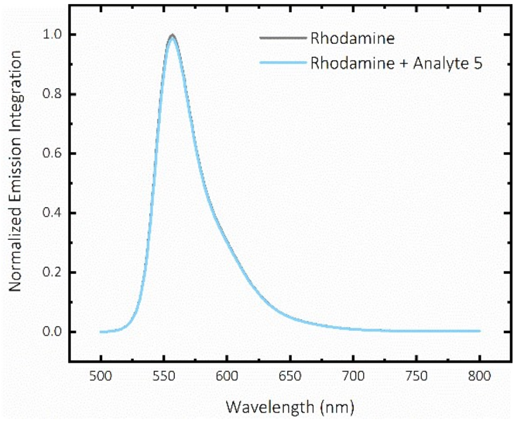

Figure S56. Fluorescence modulation of analyte **5** in the presence of 2-HPCD

No CD

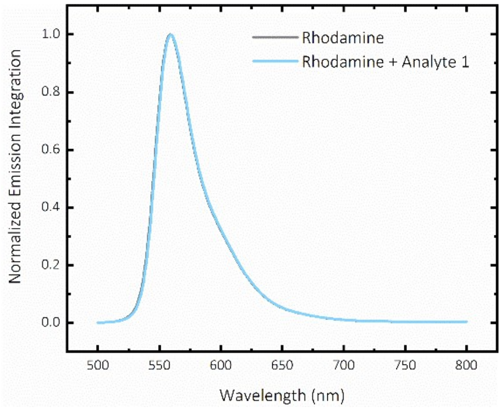

Figure S57. Fluorescence modulation of analyte **1** in the presence of no CD

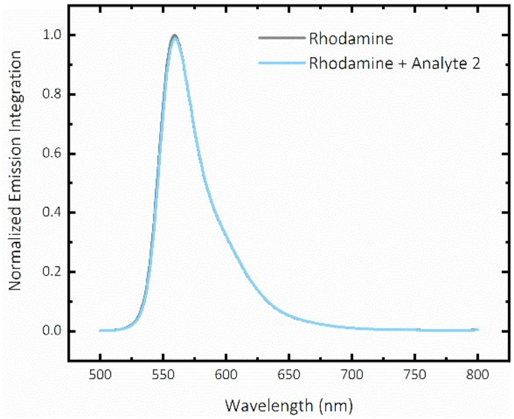

Figure S58. Fluorescence modulation of analyte **2** in the presence of no CD

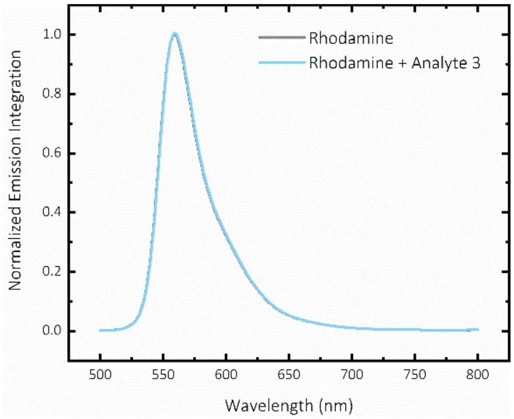

Figure S59. Fluorescence modulation of analyte **3** in the presence of no CD

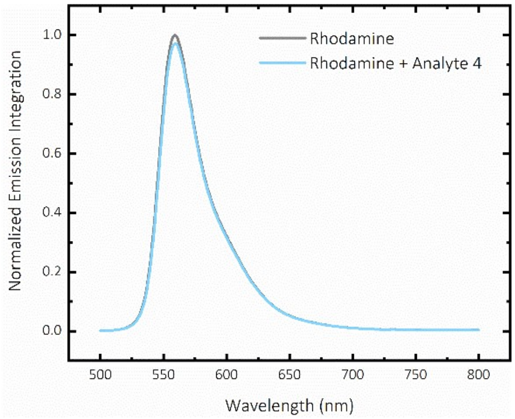

Figure S60. Fluorescence modulation of analyte **4** in the presence of no CD

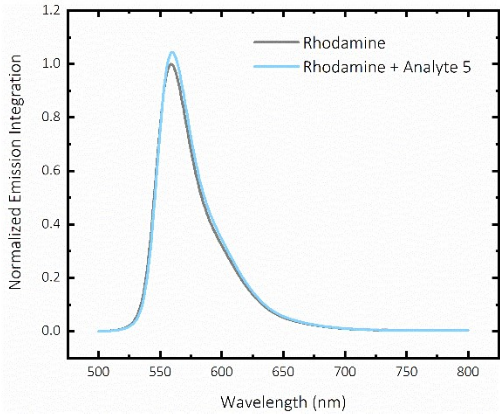

Figure S61. Fluorescence modulation of analyte **5** in the presence of no CD

Summary Figures for Combined Fluorescence Modulation Experiments using Fluorophore 6

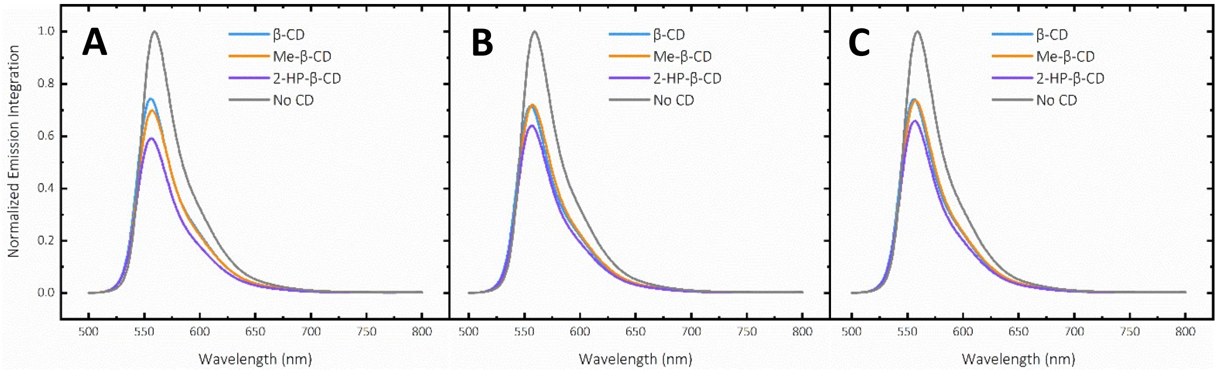

Figure S62. Fluorescence modulation of fluorophore **6** induced by: (A) 5 µL of THF (B) 10 µL of THF (C) 20 µL of THF in the presence of all hosts

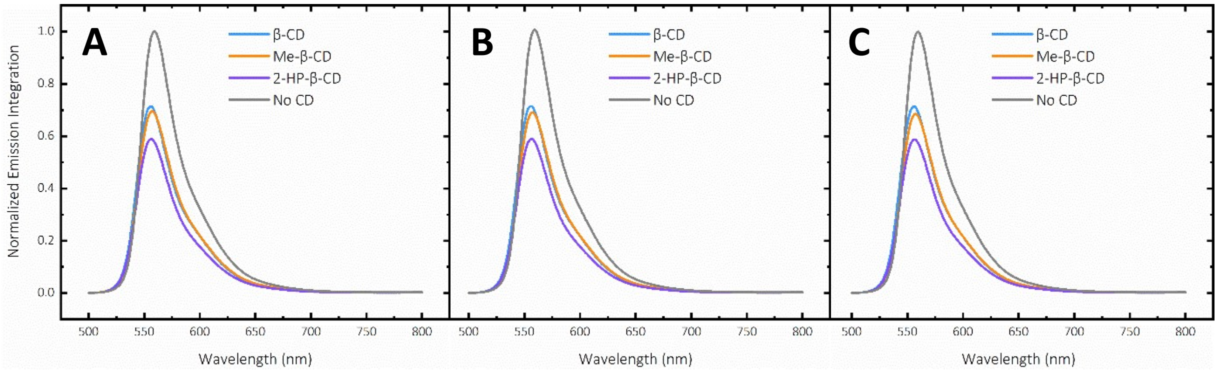

Figure S63. Fluorescence modulation of fluorophore **6** induced by: (A) 5 µL of analyte **1** (B) 10 µL of analyte **1** (C) 20 µL of analyte **1** in the presence of all hosts

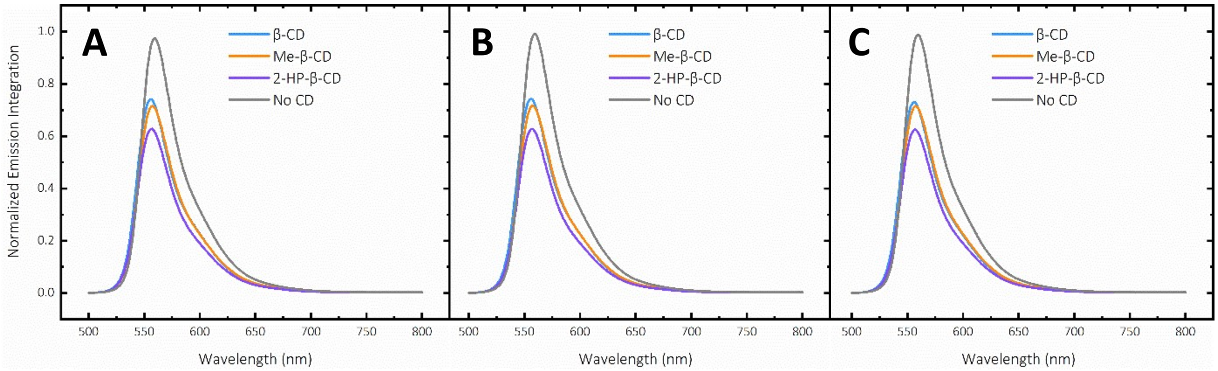

Figure S64. Fluorescence modulation of fluorophore **6** induced by: (A) 5 µL of analyte **2** (B) 10 µL of analyte **2** (C) 20 µL of analyte **2** in the presence of all hosts

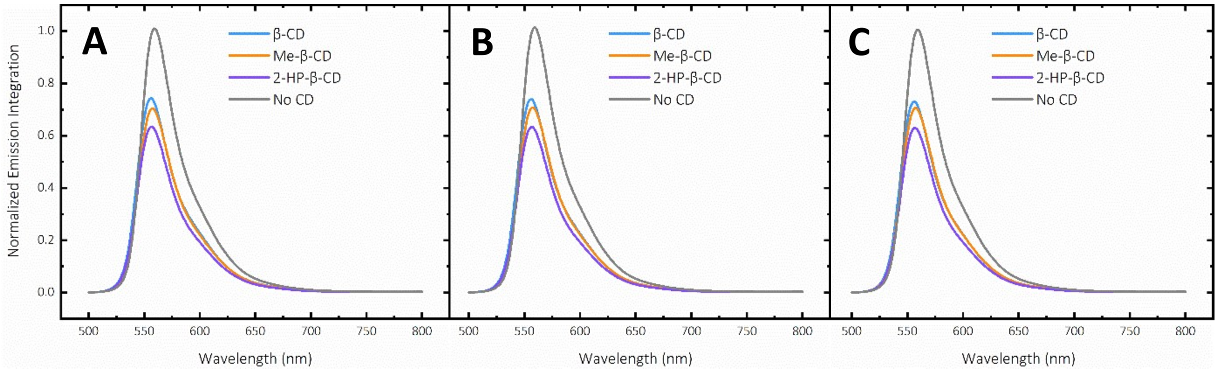

Figure S65. Fluorescence modulation of fluorophore **6** induced by: (A) 5 µL of analyte **3** (B) 10 µL of analyte **3** (C) 20 µL of analyte **3** in the presence of all hosts

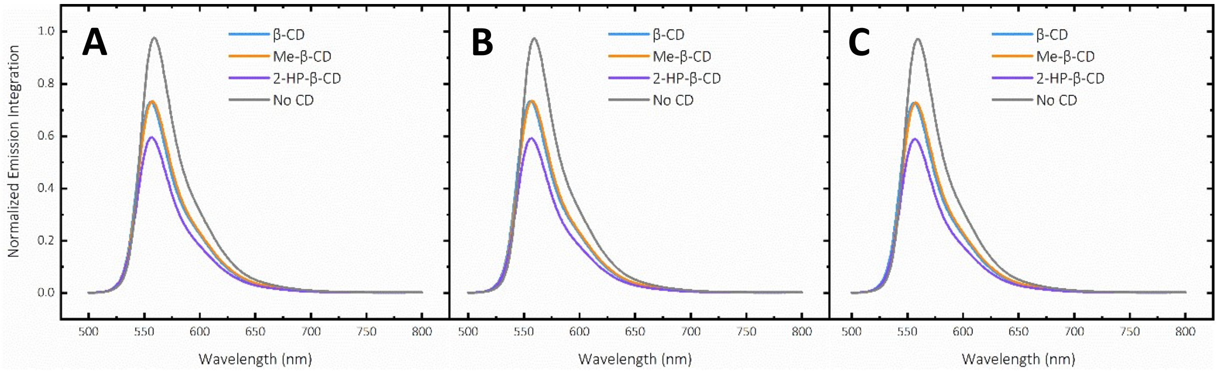

Figure S66. Fluorescence modulation of fluorophore **6** induced by: (A) 5 µL of analyte **4** (B) 10 µL of analyte **4** (C) 20 µL of analyte **4** in the presence of all hosts

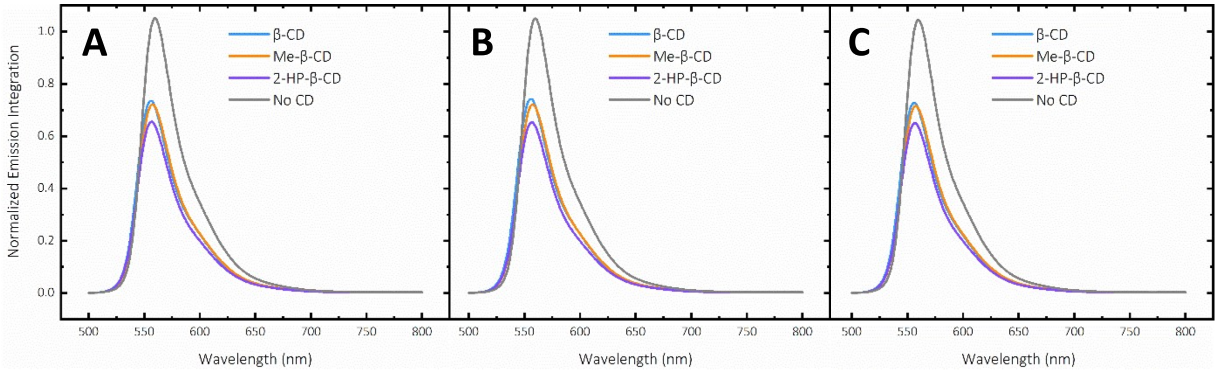

Figure S67. Fluorescence modulation of fluorophore **6** induced by: (A) 5 µL of analyte **5** (B) 10 µL of analyte **5** (C) 20 µL of analyte **5** in the presence of all hosts

Summary Figures for Limit of Detection (LOD) Experiments

Experiments were carried out with 5 µL sequential additions of analytes with fluorophore **6** in the presence of all cyclodextrin hosts.

Limits of detection calibration curves of analytes in presence of β-CD and fluorophore **6**

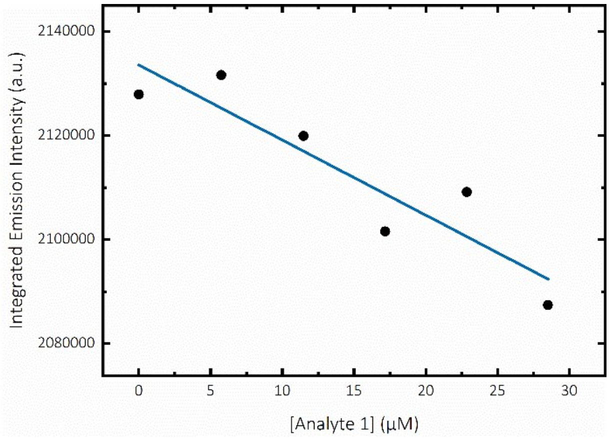

Figure S68. LOD calibration curve of analyte **1**

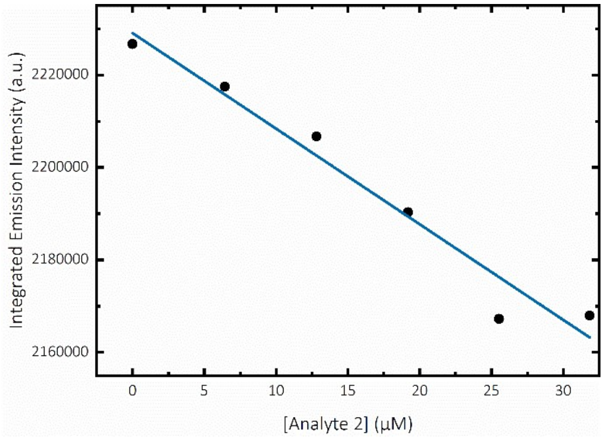

Figure S69. LOD calibration curve of analyte **2**

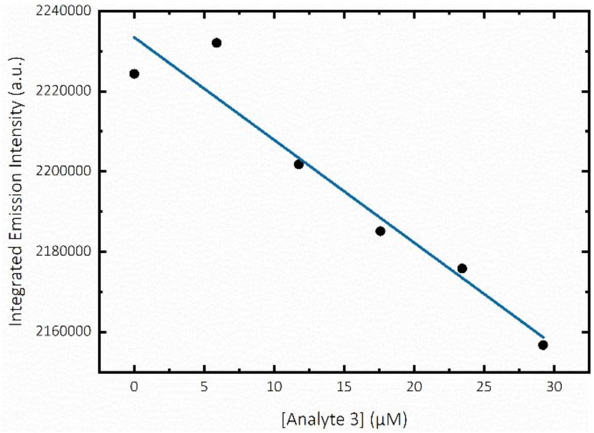

Figure S70. LOD calibration curve of analyte **3**

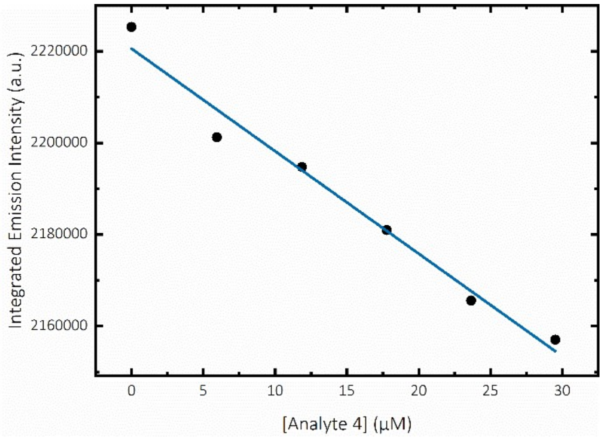

Figure S71. LOD calibration curve of analyte **4**

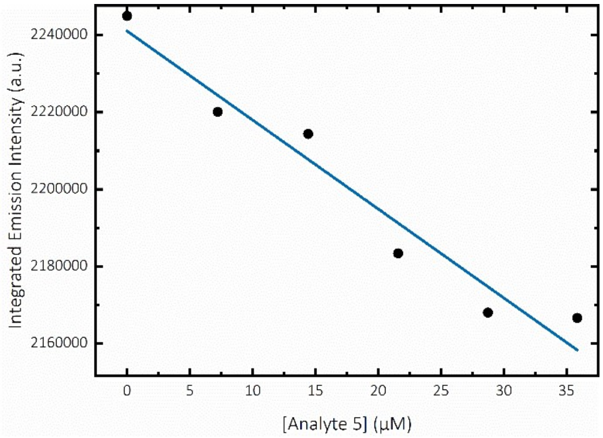

Figure S72. LOD calibration curve of analyte **5**

<u>Limits of detection calibration curves of analytes in presence of Me-β-CD and fluorophore **6**</u>

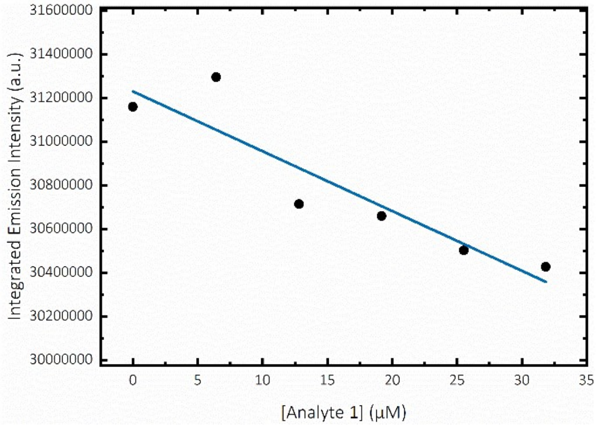

Figure S73. LOD calibration curve of analyte **1**

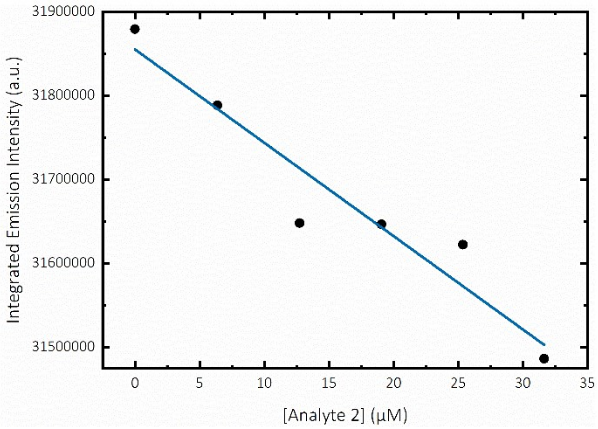

Figure S74. LOD calibration curve of analyte **2**

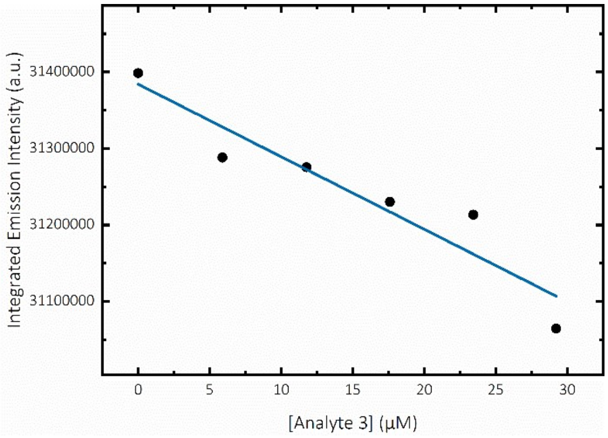

Figure S75. LOD calibration curve of analyte **3**

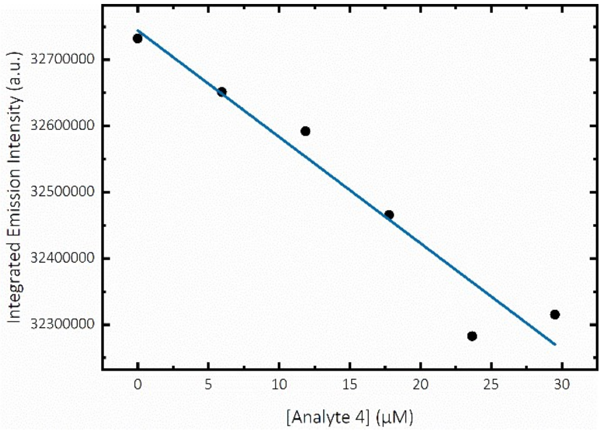

Figure S76. LOD calibration curve of analyte **4**

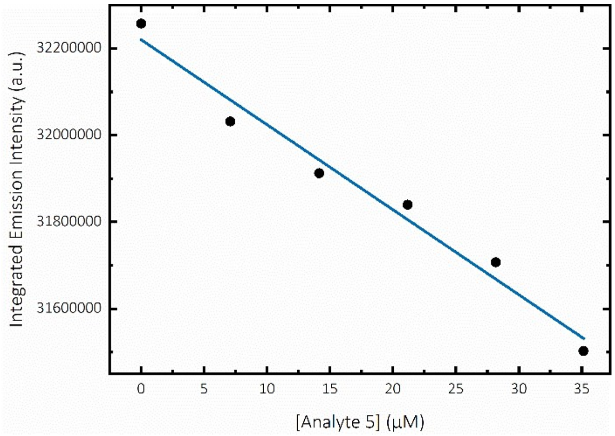

Figure S77. LOD calibration curve of analyte **5**

<u>Limits of detection calibration curves of analytes in presence of 2-HPCD and fluorophore **6**</u>

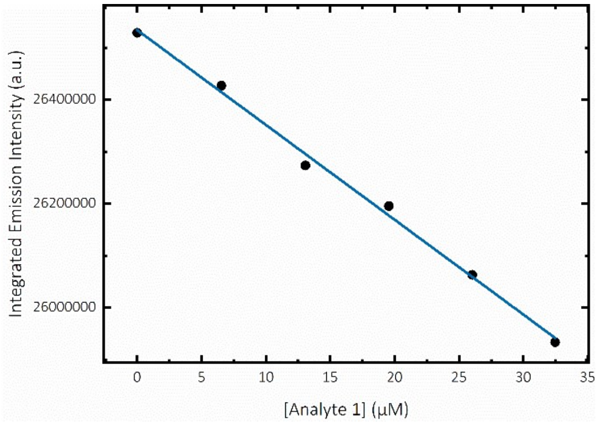

Figure S78. LOD calibration curve of analyte **1**

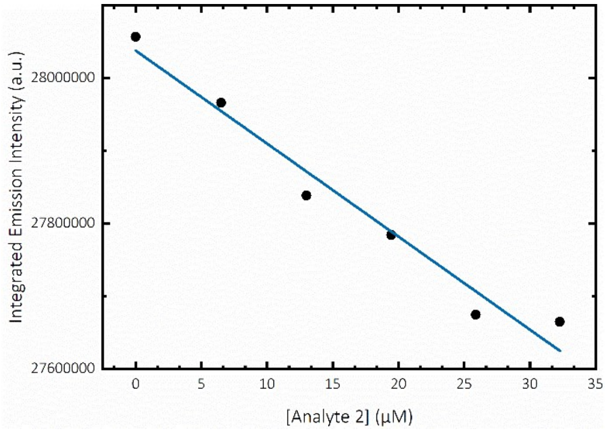

Figure S79. LOD calibration curve of analyte **2**

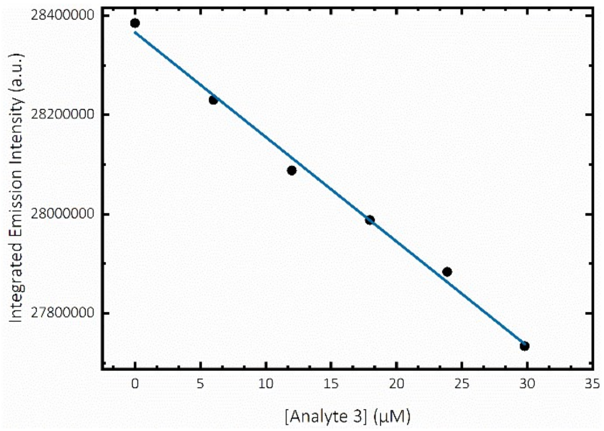

Figure S80. LOD calibration curve of analyte **3**

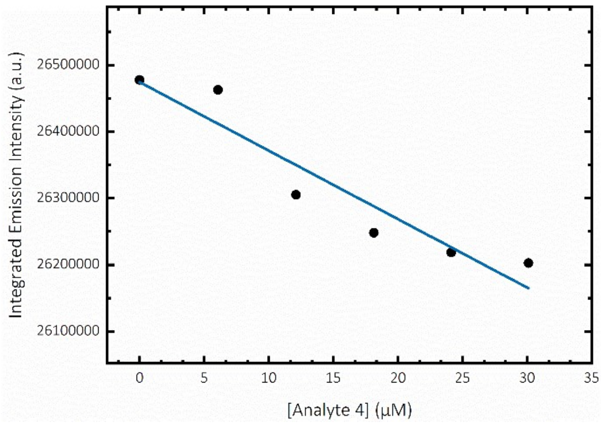

Figure S81. LOD calibration curve of analyte **4**

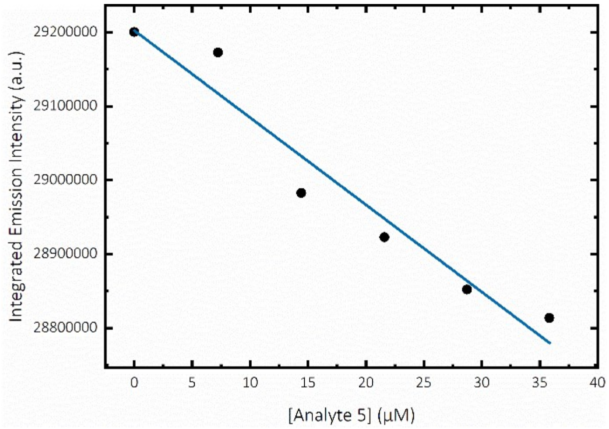

Figure S82. LOD calibration curve of analyte **5**

Summary Figures for Array Generation Experiments

<u>With 5 μL analyte additions</u>

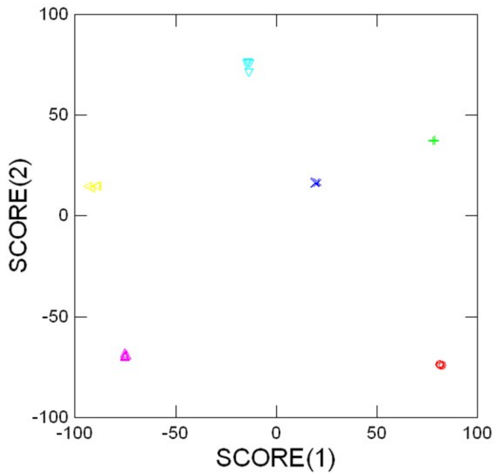

Figure S83. Linear discriminant analysis results with β-CD, Me-β-CD, and 2-HPCD as predictors

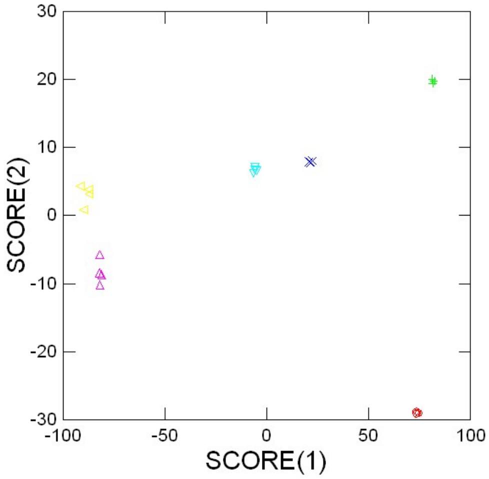

Figure S84. Linear discriminant analysis results using β-CD and Me-β-CD as predictors

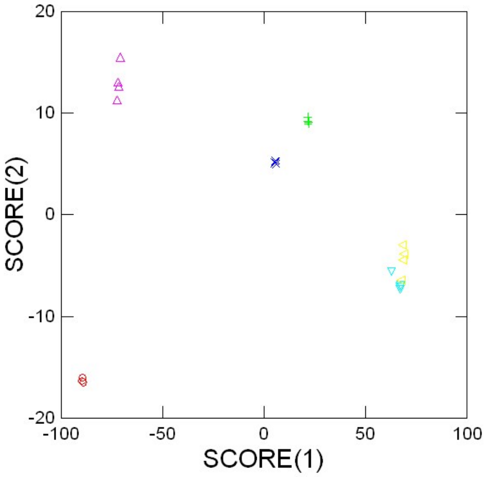

Figure S85. Linear discriminant analysis results using β-CD and 2-HPCD as predictors

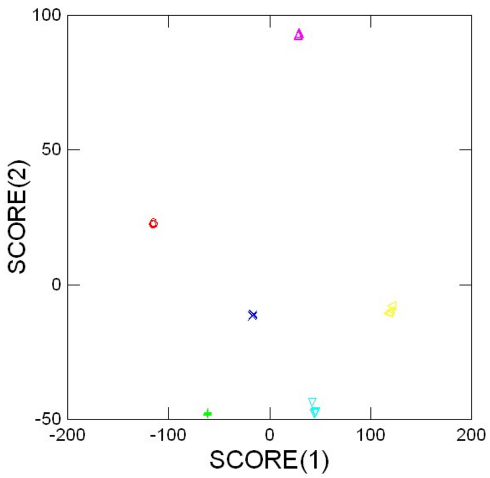

Figure S86. Linear discriminant analysis results using Me-β-CD and 2-HPCD as predictors

<u>With 10 μL analyte additions</u>

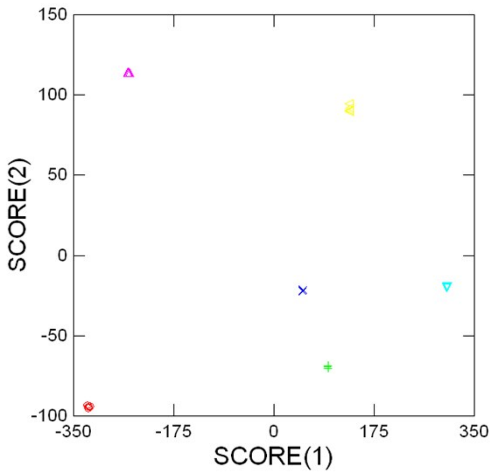

Figure S87. Linear discriminant analysis results with β-CD, Me-β-CD, and 2-HPCD as predictors

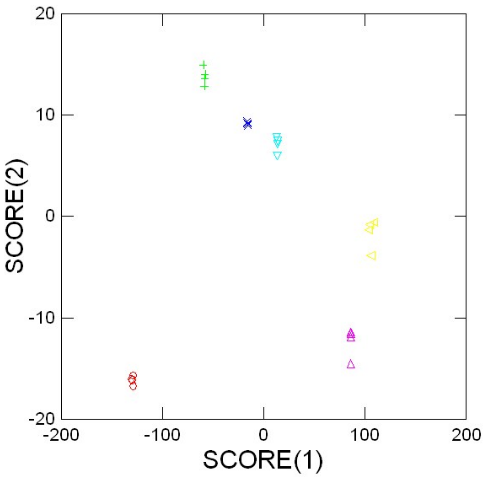

Figure S88. Linear discriminant analysis results using β-CD and Me-β-CD as predictors

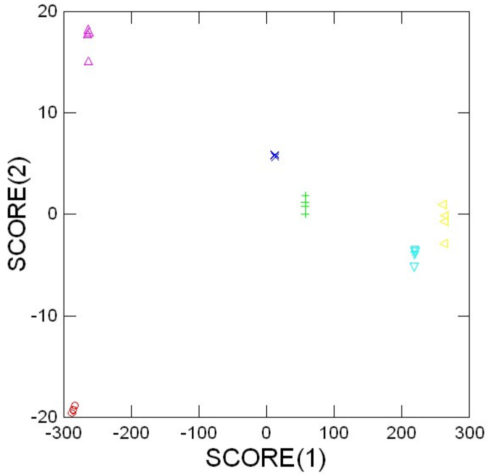

Figure S89. Linear discriminant analysis results using β-CD and 2-HPCD as predictors

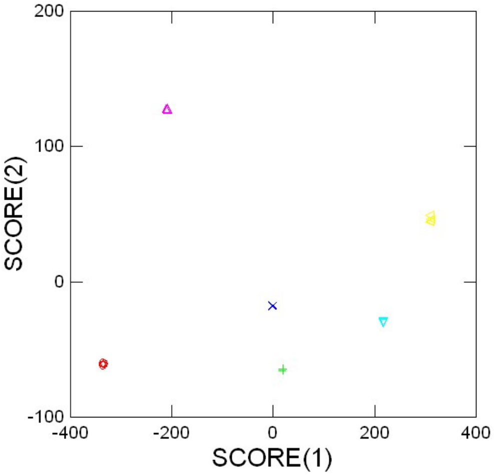

Figure S90. Linear discriminant analysis results using Me-β-CD and 2-HPCD as predictors

<u>With 20 μL analyte additions</u>

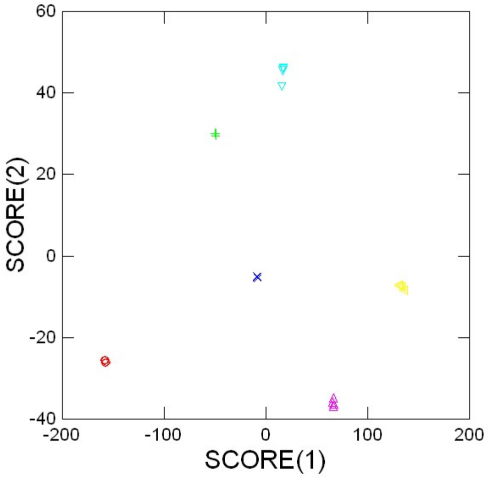

Figure S91. Linear discriminant analysis results with β-CD, Me-β-CD, and 2-HPCD as predictors

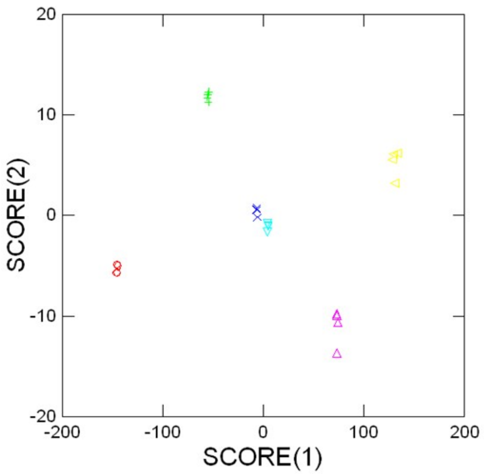

Figure S92. Linear discriminant analysis results using β-CD and Me-β-CD as predictors

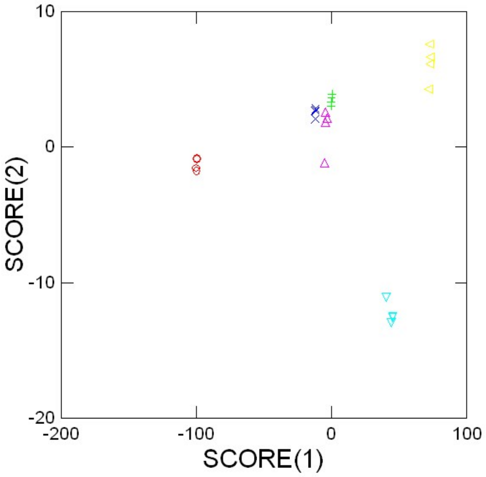

Figure S93. Linear discriminant analysis results using β-CD and 2-HPCD as predictors

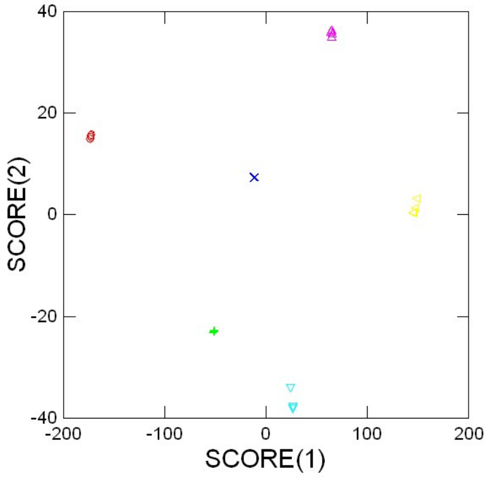

Figure S94. Linear discriminant analysis results using Me-β-CD and 2-HPCD as predictors

All Additions with THF

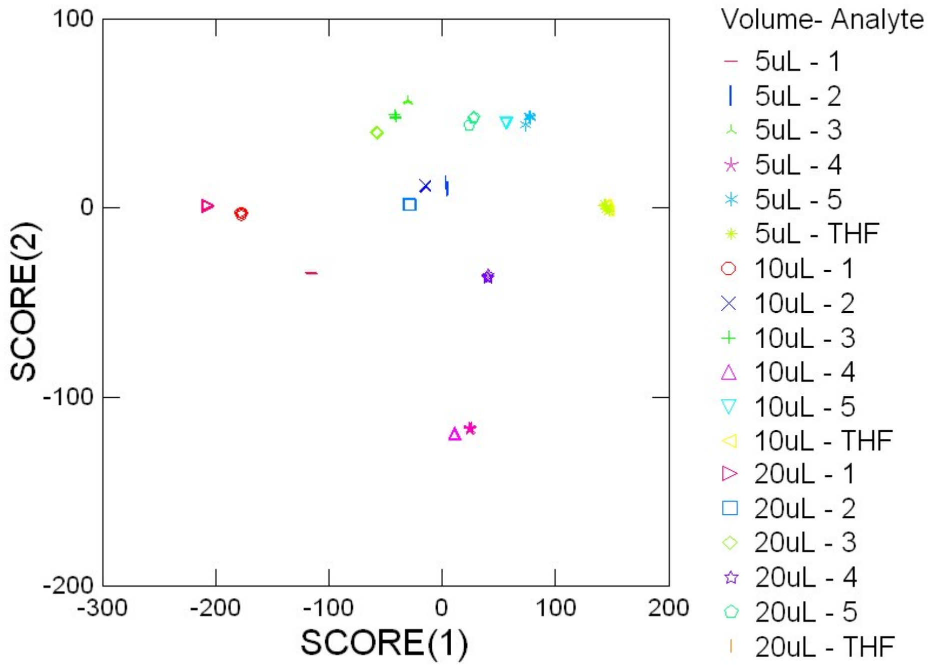

Figure S95. Linear discriminant analysis results with β-CD, Me-β-CD, and 2-HPCD as predictors

All Additions excluding THF

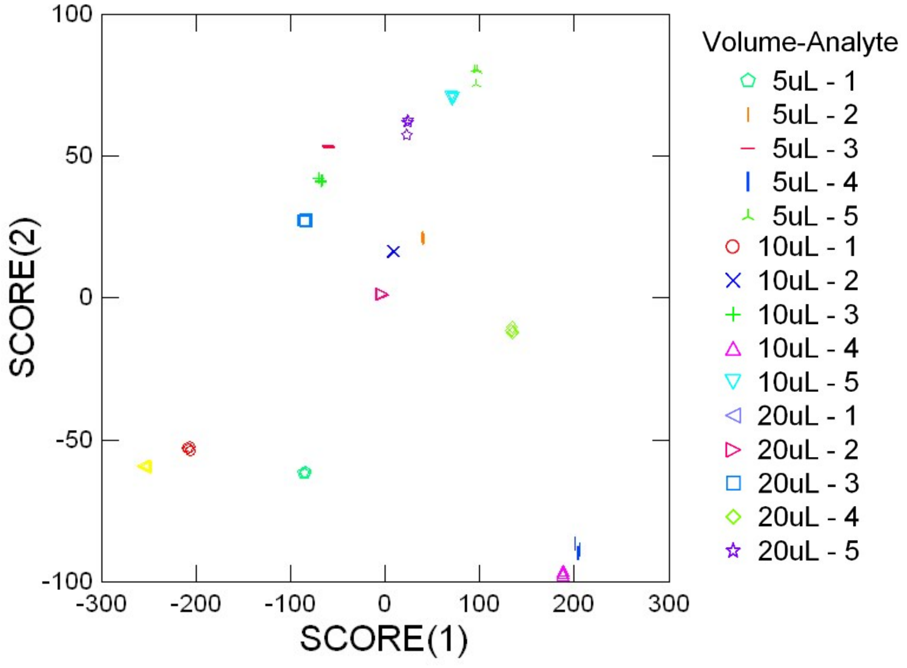

Figure S96. Linear discriminant analysis results with β-CD, Me-β-CD, and 2-HPCD as predictors

Summary Figures for Computational Experiments

<u>Spartan '18 Electrostatic Potential Map Diagrams</u>

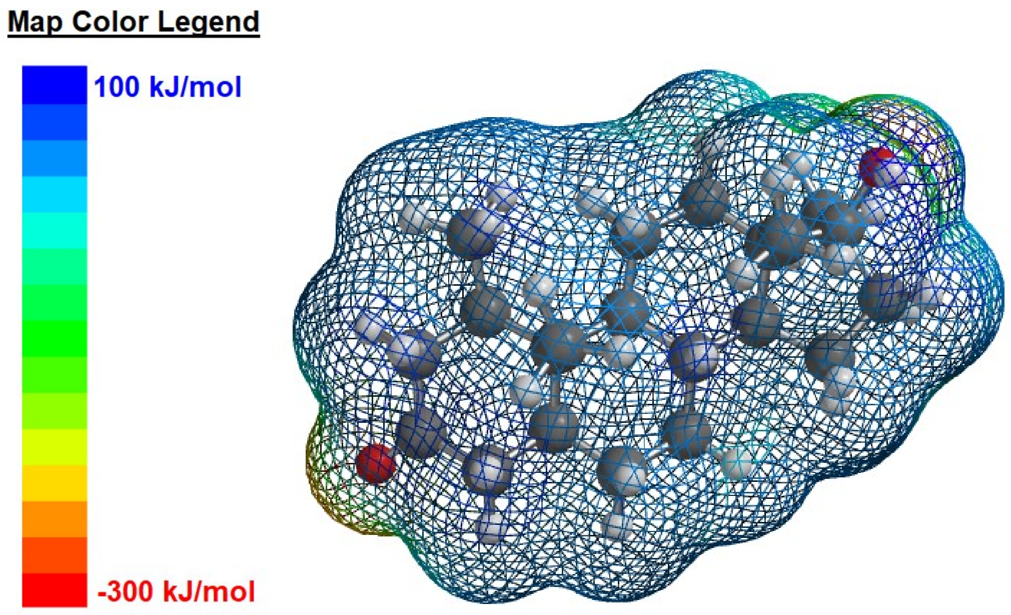

Figure S97. Electrostatic potential map of analyte **1** in the gas phase at its most stable (i.e. "ground state" configuration)

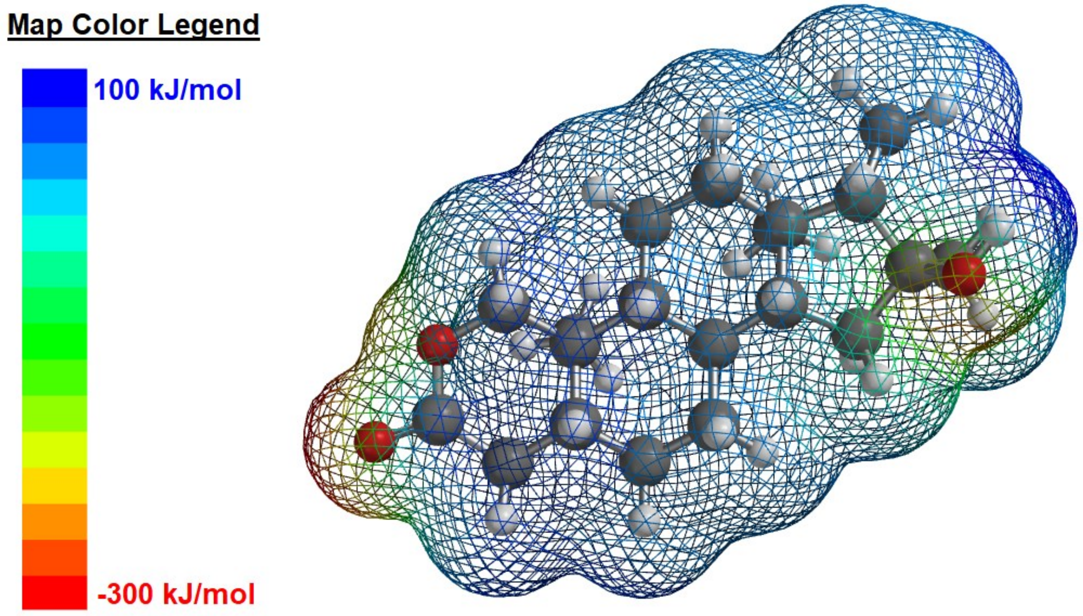

Figure S98. Electrostatic potential map of analyte **2** in the gas phase at its most stable (i.e. "ground state" configuration)

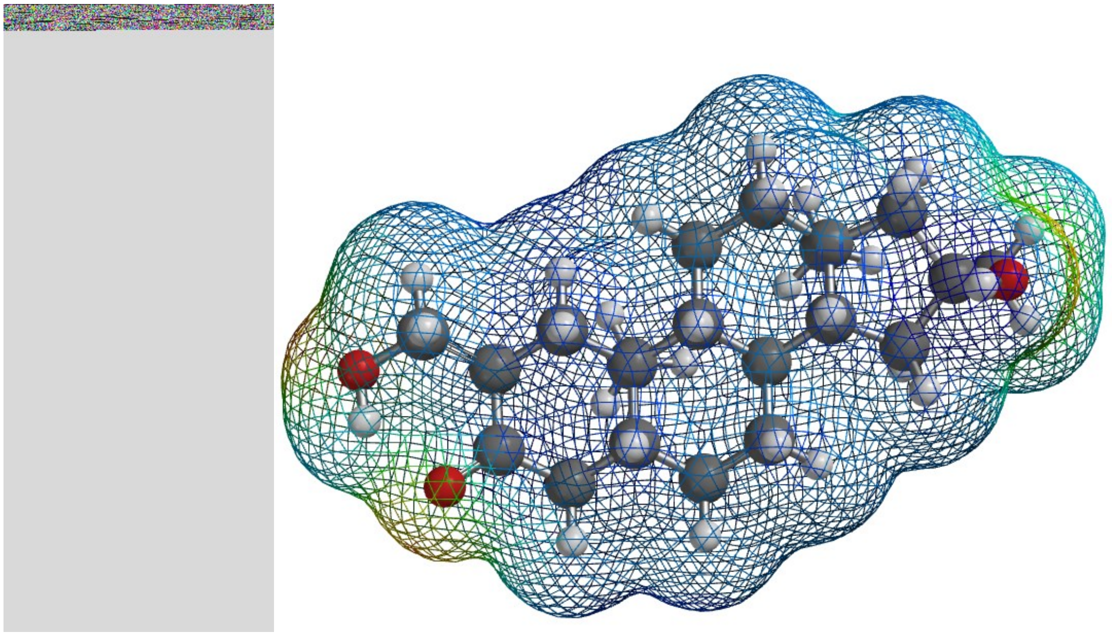

Figure S99. Electrostatic potential map of analyte **3** in the gas phase at its most stable (i.e. "ground state" configuration)

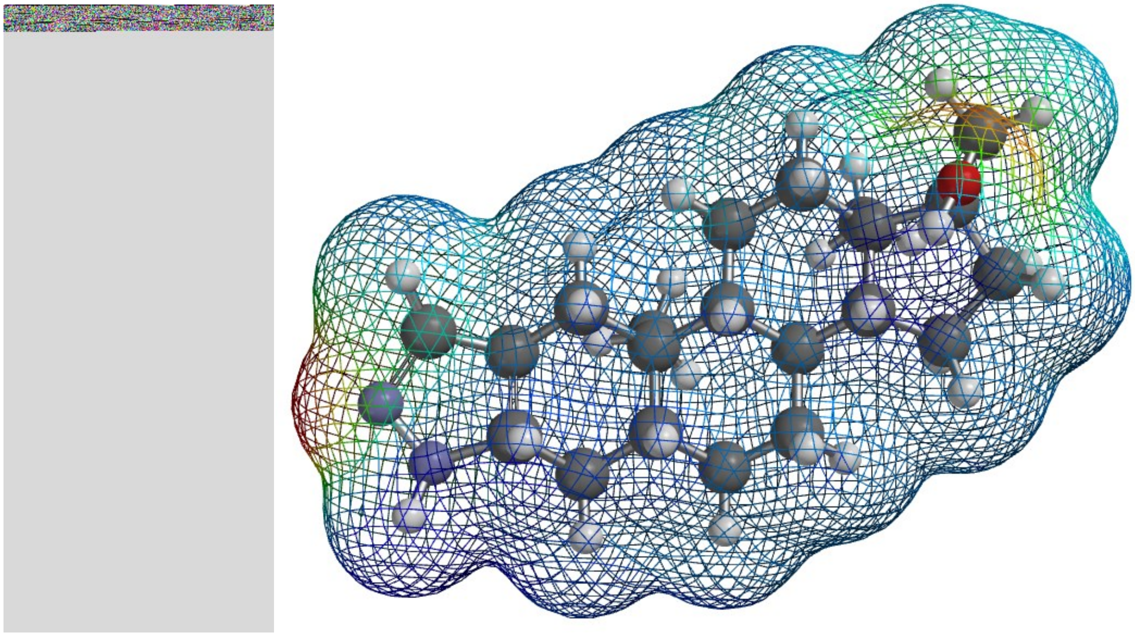

Figure S100. Electrostatic potential map of analyte **4** in the gas phase at its most stable (i.e. "ground state" configuration)

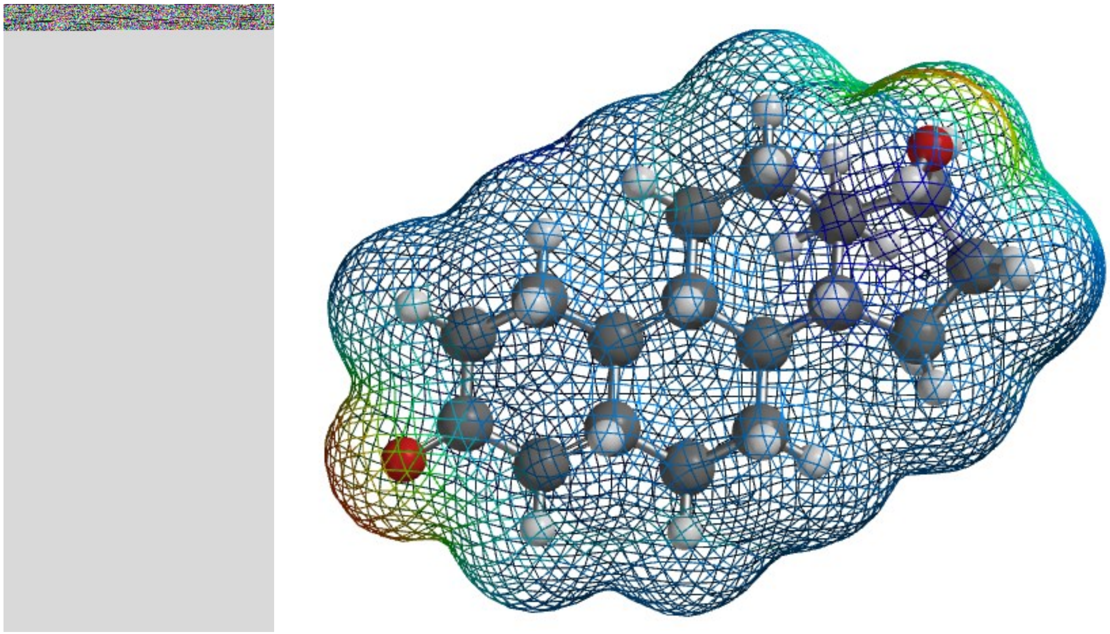

Figure S101. Electrostatic potential map of analyte **5** in the gas phase at its most stable (i.e. "ground state" configuration)

REFERENCES

(1) Loock, H.-P.; Wentzell, P. D. Detection Limits of Chemical Sensors: Applications and Misapplications. *Sensors and Actuators B: Chemical* **2012**, *173*, 157–163. https://doi.org/10.1016/j.snb.2012.06.071.

(2) Belter, M.; Sajnóg, A.; Barałkiewicz, D. Over a Century of Detection and Quantification Capabilities in Analytical Chemistry – Historical Overview and Trends. *Talanta* **2014**, *129*, 606–616. https://doi.org/10.1016/j.talanta.2014.05.018.

(3) DiScenza, D. J.; Lynch, J.; Miller, J.; Verderame, M.; Levine, M. Detection of Organochlorine Pesticides in Contaminated Marine Environments via Cyclodextrin-Promoted Fluorescence Modulation. *ACS Omega* **2017**, *2* (12), 8591–8599. https://doi.org/10.1021/acsomega.7b00991.
